# TRPM2 protects against cisplatin-induced acute kidney injury and mitochondrial dysfunction via modulating autophagy

**DOI:** 10.7150/thno.84655

**Published:** 2023-07-31

**Authors:** Binfeng Yu, Lini Jin, Xi Yao, Yi Zhang, Gensheng Zhang, Fangqin Wang, Xinwan Su, Qiuyuan Fang, Liang Xiao, Yi Yang, Lin-Hua Jiang, Jianghua Chen, Wei Yang, Weiqiang Lin, Fei Han

**Affiliations:** 1 Kidney Disease Center, The First Affiliated Hospital, Zhejiang University School of Medicine; Institute of Nephrology, Zhejiang University; Key Laboratory of Kidney Disease Prevention and Control Technology, Zhejiang Province; Zhejiang Clinical Research Center of Kidney and Urinary System Disease, Hangzhou 310003, China.; 2 Department of Infectious Disease, Sir Run Run Shaw Hospital, Zhejiang University School of medicine, Hangzhou 310003, China.; 3 International Institutes of Medicine, The Fourth Affiliated Hospital, Zhejiang University School of Medicine, Yiwu 322000, China.; 4 Department of Biophysics, and Department of Neurology of the Fourth Affiliated Hospital, Zhejiang University School of Medicine, Hangzhou 310003, China.; 5 The Children's Hospital, Zhejiang University School of medicine, Hangzhou 310003, China.; 6 Sino-UK Joint Laboratory of Brain Function and Injury of Henan Province, and Department of Physiology and Pathophysiology, Xinxiang Medical University, P.R. China.; 7 A4245-Transplantation, Immunology and Inflammation, Faculty of Medicine, University of Tours, France.; 8 School of Biomedical Sciences, Faculty of Biological Sciences, University of Leeds, UK.

**Keywords:** TRPM2, autophagy, mitochondria, cisplatin, acute kidney injury

## Abstract

**Background:** Cisplatin is a widely used anti-tumor agent but its use is frequently limited by nephrotoxicity. Transient receptor potential melastatin 2 (TRPM2) is a non-selective cation channel which is generally viewed as a sensor of oxidative stress, and increasing evidence supports its link with autophagy, a critical process for organelle homeostasis.

**Methods:** Cisplatin-induced cell injury and mitochondrial damage were both assessed in WT and *Trpm2-*knockout mice and primary cells. RNA sequencing, immunofluorescence staining, immunoblotting and flowcytometry were applied to interpret the mechanism of TRPM2 in cisplatin nephrotoxicity.

**Results:** Knockout of TRPM2 exacerbates renal dysfunction, tubular injury and cell apoptosis in a model of acute kidney injury (AKI) induced by treatment with cisplatin. Cisplatin-caused tubular mitochondrial damage is aggravated in TRPM2-deficient mice and cells and, conversely, alleviated by treatment with Mito-TEMPO, a mitochondrial ROS scavenger. TRPM2 deficiency hinders cisplatin-induced autophagy via blockage of Ca^2+^ influx and subsequent up-regulation of AKT-mTOR signaling. Consistently, cisplatin-induced tubular mitochondrial damage, cell apoptosis and renal dysfunction in TRPM2-deficient mice are mitigated by treatment with a mTOR inhibitor.

**Conclusion:** Our results suggest that the TRPM2 channel plays a protective role in cisplatin-induced AKI via modulating the Ca^2+^-AKT-mTOR signaling pathway and autophagy, providing novel insights into the pathogenesis of kidney injury.

## Introduction

Acute kidney injury (AKI) is a common clinical condition defined by a rapid decline of glomerular filtration function, mainly caused by hypotension, dehydration, sepsis, nephrotoxins and renal ischemia-reperfusion 1-4. 4. The incidence of AKI during hospitalization is estimated to be 10%-15% 5. Cisplatin is a highly effective agent for treating multiple solid tumors, but its dose-dependent nephrotoxicity frequently curtails its clinical use 6, 7. It is estimated that approximately 30% of adult patients treated with cisplatin experienced AKI and a majority of patients who survived for at least 5 years suffered a permanent decline in glomerular filtration function 8. To date, the mechanisms underlying cisplatin-induced nephrotoxicity remains poorly understood. Nonetheless, growing evidence supports that mitochondrial dysfunction plays a pivotal role in the development of AKI caused by cisplatin 2, 9 and other conditions such as ischemia-reperfusion 1 and sepsis 3. Mitochondria are the central cellular hub of energy production and thus participate in a variety of physiological processes. Disruption of mitochondria not only compromises production of energy but also increases production of reactive oxygen species (ROS) and induces release of cytochrome (Cyt)-c and mitochondrial DNA (mtDNA), triggering cell apoptosis and inflammation 10-12. Therefore, mitochondria are a potential therapeutic target for treatment of AKI.

Autophagy is a lysosome-mediated degradation process that recycles cellular components including proteins, lipids and organelles and thus is important in maintaining cellular homeostasis. Previous studies have shown that autophagy occurs at an early stage of cisplatin-induced AKI and plays a crucial role in protecting against kidney injury 13, and that renal proximal tubule-specific knockout of *Atg7* or *Atg5* expression impaired cisplatin-induced autophagy in tubules and aggravated cell apoptosis and kidney damage 14, 15. In addition, induction of transcription factor EB-mediated autophagy by trehalose ameliorated cisplatin-induced mitochondrial damage and kidney injury 9. These findings support the importance of autophagy in AKI pathogenesis but the current understanding is still limited.

Transient receptor potential melastatin 2 (TRPM2) is the second member of the melastatin subfamily of transient receptor potential superfamily. As a Ca^2+^-permeable none-selective cation channel, TRPM2 is expressed in a variety of organs and tissues, including brain, heart, liver, kidney and pancreatic islet 16. Extracellular signals such as ROS and tumor necrosis factor-α can activate the TRPM2 channel, by promoting intracellular production of adenosine diphosphate-ribose (ADPR) that specifically binds to and activates the TRPM2 channel 17, 18, and contribute to oxidative damage and cell death 16. Our previous studies revealed the structural and functional basis of selectivity filter in human TRPM2 channel 18 and identified cADPR as an activator to TRPM2 channel via its specific interactions with the ADPR Binding Pocket 19. A number of studies support that TRPM2 plays a critical role in multiple pathological conditions, such as post-ischemic brain injury 20, diabetes 21 and liver damage 22. Nonetheless, some studies have highlighted the physiological significance of TRPM2-mediated Ca^2+^ signaling in embryonic neurogenesis 23, radiation-induced DNA damage response 24, and bacterial clearance in *Escherichia coli* sepsis 25, and plays a protective role in cardiac ischemic injury 26 and pneumonic bacterial infection 27. Moreover, TRPM2 deficiency disturbed mitochondrial homeostasis and autophagic process in leukemia and gastric cancer cells, rendering them more susceptible to chemotherapy 28, 29. There is evidence that the TRPM2 channel mediates ischemic kidney injury 30. However, the role of TRPM2 in AKI caused by other etiologies such as treatment with cisplatin is still unknown.

In this study, we investigated the role of TRPM2 in cisplatin-induced AKI pathogenesis, focusing on its role in mediating cisplatin-induced effects on mitochondrial function. Our results from *in vivo* and *in vitro* experiments consistently show that TRPM2 promotes cisplatin-induced autophagy to maintain mitochondrial homeostasis through inhibiting the protein kinase B (AKT)-mammalian target of rapamycin (mTOR) signaling pathway and protects against cisplatin-induced kidney injury.

## Methods

### Cell preparation and treatment

Primary mouse embryonic fibroblasts (MEFs) were obtained from embryos at E13.5 and cultured in DMEM (Gibco, C11995500BT) supplemented with 10% FBS and 1% penicillin/streptomycin at 37℃ in an incubator with 5% CO_2_ as previously described 31. Primary culture of mouse renal tubular epithelial cells (mRTECs) was performed as previously described 32. Briefly, kidneys were harvested from 3- to 5-week-old male mice, followed by cutting into pieces and digesting with 2 mg/mL collagenase I (Worthington, LS004196) at 37 ℃ for 30 min. Cells were filtered through a 100-μm strainer and cultured in RPMI 1640 (Sigma-Aldrich, R-8758) supplemented with 10% FBS, 20 ng/mL epidermal growth factor (Peprotech, AF-100-15), ITS-X (Gibco, 51500-056) and 1% penicillin/streptomycin. Staining with fibroblast marker Vimentin and tubular epithelial marker AQP-1 was used for the verification of isolated MEFs and mRTECs, respectively Figure S1A-B). HK-2, HCT116, SW480 and ACHN were purchased from American Type Culture Collection, and cultured in DMEM/F12 (Sigma-Aldrich, D8437) supplemented with 10% FBS for HK-2 and RPMI 1640 supplemented with 10% FBS for other cell types.

To induce apoptosis, MEFs and HK-2 cells were treated with 20 µM cisplatin (Selleck, S1166) and mRTECs with 5 μM cisplatin for 24 h, respectively. Clotrimazole (CLT, 10 μM, Target Mol, T0506), 2-APB (20 μM, Sigma-Aldrich, D9754), ADPR (100 μM, MedChemExpress, HY-100973A), chloroquine (CQ, 10 μM, MedChemExpress, HY-17589A), Mito-TEMPO (200 nM, MedChemExpress, HY-112879), BAPTA-AM (5 μM, MedChemExpress, HY-100545), 3-methyladenine (3-MA, 5 mM, Selleck, S2767), VIII (5 μM, Beyotime, SF2784) and rapamycin (50 nM, MedChemExpress, HY-10219) were added to cell culture medium 1 h before incubation with cisplatin. For induction of autophagy, cells were treated with H_2_O_2_ (Sinopharm Chemical Reagent, 10011218) at indicated concentrations for 3 h. To evaluate autophagic flux, MEFs were transfected with lentiviral vector carrying mRFP-GFP-LC3 (HANBIO, HB-LP210 0001). To overexpress TRPM2 in mRTECs, cells were infected with adenovirus encoding mouse TRPM2 (Adv-TRPM2, purchased from OBiO Technology) for 24 h before subsequent interventions. All experiments were performed in triplicate or in quadruplicate.

### Animals

C57BL/6 WT mice were purchased from Shanghai SLAC Laboratory. *Trpm2^-/-^* transgenic mice were introduced from the University of Leeds and genetic validation was shown in Figure S1C as previously described (33. These mice express a TRPM2 protein that is lack of transmembrane domains 3 and 4 due to deletion of exons 17 and 18 and does not form functional channel. All mice were housed in a specific-pathogen-free facility with a 12-h light/dark cycle. All animal experiments were approved by the Committees for Animal Experiments of Zhejiang University (approval number, ZJU20220301) and performed following the policies of Zhejiang University.

### Cisplatin-induced AKI models

To induce AKI, 8- to 10-week-old WT and *Trpm2^-/-^* male mice were subjected to a single intraperitoneal (i.p.) injection of 18 mg/kg cisplatin. The mice were euthanized at 72 h and the blood and kidney tissues were harvested for further analysis. In some experiments, mice were either injected i.p. with 7 mg/kg Mito-TEMPO or an equal volume of PBS once daily, starting 7 days before cisplatin injection and continuing until the day before the mice were sacrificed. In some other experiments, mice were either injected i.p. with 1 mg/kg rapamycin or an equal volume of vehicle (5% DMSO+30% PEG400) 1 h prior to and 1 day after cisplatin injection as previously described 15, 34. For inhibition of TRPM2 activity, mice were injected i.p. with 10 mg/kg 2-APB 1 h prior to cisplatin injection with minor modification on the method from a previous study 35. For inhibition of AKT activity, mice were injected i.p. with 50 mg/kg VIII 1 h prior to cisplatin injection.

### Renal function, histopathology and immunohistochemistry analysis

Renal function was assessed by measuring serum creatinine using the FUJIDRI-CHEM 7000i biochemistry analyzer (FUJIFILM, Tokyo, Japan). Kidney tissues were fixed in 4% paraformaldehyde (PFA), embedded in paraffin, and sliced into 4-μm-thick sections for Periodic Acid-Schiff (PAS) staining. Tubular injury was semi-quantitatively scored by the percentage of damaged tubules and histological injury: 0, no damage; 1, < 25%; 2, 25%-50%; 3, 50%-75%; 4, > 75% 36. The assessment was performed by two pathologists blinded to the experiments.

For immunohistochemistry, kidney sections from mice or human were immersed in EDTA/EGTA buffer (pH 9.0) and heated to boiling for antigen retrieval. 8-OHdG (1:100, Santa Cruz, sc-66036) or TRPM2 (1:100, ET1703-34, Huabio) antibody was incubated at 37 °C for 3 h. The images were captured using a Leica DM4000 microscope.

### Immunofluorescence staining

For immunofluorescence, PFA-fixed frozen kidney sections or cells were permeabilized with 0.3% Triton X-100 for 20 min and blocked with 5% bovine serum albumin for 1 h. After washed with PBS, the slides were incubated with primary antibodies targeting Calnexin (1:100, Santa Cruz, sc-23954), kidney injury marker-1 (Kim-1, 1:500, R&D Systems, AF1817), LC3B (1:200, Sigma-Aldrich, L7543), lysosomal associated membrane protein 1 (LAMP1, 1:100, Santa Cruz, sc-19992), LAMP2 (1:500, Proteintech, 66301-1-Ig), TPRM2 (1:100, Abcam, ab240540; 1:100, Bethyl Laboratories, A300-414A), TOM20 (1:500, Proteintech, 11802-1-AP), Vimentin (1:200, Huabio, HA500437) or AQP-1 (1:100, Huabio, ET1703-34), respectively or in combination at 4 ℃ overnight, followed by incubation with corresponding secondary antibodies for 1 h. Brush borders of proximal tubules were labelled by *Lotus tetragonolobus lectin* (LTL, 1:200, Vector Laboratories, B-1325), in combination with Cy3-conjugated streptavidin (1:500, Vector Laboratories, SA-1300). DAPI was used for nuclear staining. All fluorescence images were captured using a confocal microscope (Nikon A1). The numbers of positive tubules or cells from 5 randomly selected fields were averaged for each kidney sample.

### Dihydroethidium (DHE) staining

DHE staining was performed to determine the level of ROS in kidney tissues as previously described 37. Briefly, kidneys were rapidly harvested and freshly frozen in optimal cutting temperature (OCT) compound and sliced into 8-μm-thick slices. The kidney slices were incubated with 20 μM DHE (Beyotime, S0063) at room temperature in the dark for 30 min before counterstained with DAPI. Images were captured using a confocal microscope (Nikon A1).

### Transmission electron microscopy (TEM)

Kidney cortex tissues in 1 mm^3^ and collected cells were firstly fixed with 2.5% glutaraldehyde and 1% osmic acid and then fixed and dyed with 2% uranyl acetate, followed by dehydration in ethanol and acetone. Tissues and cells were embedded and polymerized at 37℃ overnight and sliced into ultrathin sections, stained with uranyl acetate and lead citrate. Images were captured using a transmission electron microscope (Philips).

### Measurement of mitochondrial membrane potential (MMP)

The MMP was determined by JC-1 staining (MedChemExpress, HY-15534) following manufacturer's instructions. Briefly, cells were incubated with 2 μM JC-1 for 20 min at 37°C in the dark, washed with PBS, and analyzed by a flow cytometer with 488-nm excitation. The relative MMP was calculated by the ratio of J-aggregate/monomer, i.e. red to green fluorescence intensity emitted at 590 and 520 nm, respectively.

### Measurement of mitochondrial ROS (mtROS)

The level of mtROS in living MEFs was detected by MitoSOX (Invitrogen, M36008). Briefly, cells were incubated in 5 μM MitoSOX at 37 ℃ for 10 min before harvested and detected by flow cytometry at 550-nm excitation.

### Mitochondrial morphology analysis

Cells were incubated with 200 nM MitoTracker Red CMXRos (Invitrogen, M7512) at 37°C for 30 min. Cells were rinsed with PBS twice and detected using a confocal microscope (Nikon A1) at 550-nm excitation.

### Measurement of intracellular Ca^2+^

For the measurement of intracellular Ca^2+^, cells were loaded with 4 μM Fluo-4 AM (Yeasen, 40704ES50) in Hank's Balanced Salt Solution (HBSS) buffer in combination with 0.05% Pluronic F-127 at 37°C for 30 min. Cells were rinsed with HBSS three times and detected using a confocal microscope (Nikon A1) at 488-nm excitation.

### Measurement of mitochondrial Ca^2+^

For the measurement of mitochondrial Ca^2+^, cells were loaded with 4 μM Rhod-2 AM (Yeasen, 40776ES50) in HBSS buffer in combination with 0.05% Pluronic F-127 at 37°C for 30 min. Cells were rinsed with HBSS three times and detected under a confocal microscope (Nikon A1) at 550-nm excitation.

### Mitochondrial isolation

Isolation of mitochondria from kidney tissues was performed using Tissue Mitochondrial Extraction Kit (Beyotime, C3606) following manufacturer's instructions. Isolation of mitochondria in cells was performed using Cell Mitochondrial Extraction Kit (Beyotime, C3601) following manufacturer's instructions. The mitochondrial and cytoplasmic fractions were isolated through differential centrifugation and lysed for further immunoblotting analysis.

### RNA-seq profiling

RNA-sequencing was performed in Novogene on Illumina Hiseq 2500 platform as previously described 38. Briefly, RNA was isolated from kidney tissues using Trizol. Sequencing libraries were constructed using NEBNext Ultra II RNA Library Prep Kit for Illumina (NEB, E7770). Principal Component Analysis (PCA) was conducted based on Fragments Per Kilobase Million from each sample. Differentially expressed genes were defined as |log2(FoldChange)| > 1, *P* value < 0.05. Kyoto Encyclopedia of Genes and Genomes (KEGG) enrichment analysis was performed by clusterProfiler software. Gene Set Enrichment Analysis (GSEA) was conducted based on the fold change of differentially expressed genes.

### Immunoblotting

Kidney tissues and cells were lysed in RIPA lysis buffer (Millipore, 20-188). Protein concentrations were determined using a Bradford assay (Beyotime, P0006C). 15-20 µg proteins were separated by SDS-PAGE and transferred to polyvinylidene difluoride membranes. After blocked in 5% fat-free milk for 1 h, membranes were incubated in the following primary antibodies at 4℃ overnight: anti-LC3B (1:1000, L7543) from Sigma-Aldrich; anti-Bcl2 (1:1000, 26593-1-AP); anti-caspase-3 (1:1000, 19677-1-AP), anti-Cyt-C (1:1000, 10993-1-AP), anti-GAPDH (1:5000, 60004-1-Ig) from Proteintech; anti-p62 (1:1000, A19700), anti-AMPKα1 (1:1000, A1229), anti- adenovirus E1B 19-kDa-interacting protein 3 (BNIP3, 1:1000, A19593) from ABclonal; anti-DRP1 (1:1000, #8570), anti-TOM20 (1:1000, #42406), anti-phospho-mTOR (Ser2448, 1:1000, #5536), anti-mTOR (1:1000, #2983), anti-phospho-AKT (Ser473, 1:1000, #9271), anti-AKT (1:1000, #4691), anti-phospho-p70S6K (Thr389, 1:1000, #9234) from CST; anti-BAX (1:1000, D220073) from BBI; anti-phospho-Beclin1 (Ser295, 1:1000, ab183313), anti-VDAC (1:1000, ab14734) from Abcam; anti-TRPM2 (1:1000, PA1-46473) from Invitrogen; anti-phospho-AMPKα (Thr172, 1:1000, AA393) from Beyotime; anti-TRPM2 (1:1000, A300-414A) from Bethyl Laboratories; anti-Beclin1 (1:1000, AP6020) from Bioworld; anti-PINK1 (1:1000, DF7742) from Affinity Biosciences. Image J software was used to quantify proteins.

### Cell viability assay

Cell viability was determined using the CCK-8 assay (MedChemExpress, HY-K0301) following manufacturer's instructions. Briefly, cells were seeded onto 96-well plates at a density of 4×10^3^ cells/well. Cells were treated with cisplatin at indicated concentrations for 24 h and subsequently incubated with CCK-8 reagent at 37℃ for 3 h. The absorbance at 450 nm was measured by a microplate reader (SpectraMax M5/M5e).

### Quantification of mtDNA

Total DNA was isolated from kidney tissues using the Universal Genomic DNA Purification Mini Spin Kit (Beyotime, D0063) following manufacturer's instructions. The mtDNA level was indicated by the ratio of mtDNA to nuclear DNA. Quantitative PCR was performed to amplify *Nd2* gene from mitochondrial genome and *Gapdh* gene from nuclear genome 39. Primer sequences used are as follows: *Gapdh* forward, 5'-CCTGCACCACCAACTGCTTAG-3'; *Gapdh* reverse, 5'-GTGGATGCAGGGATGTTC-3'; *Nd2* forward, 5'-CCCATTCCACTTCTGATTACC-3'; *Nd2* reverse, 5'-ATGATAGTAGAGTTGAGTAGCG-3'.

### Analysis of apoptosis

Apoptosis in kidney tissues was examined using the *In situ* Cell Apoptosis Detection Kit (G1507, Servicebio) according to manufacturer's instructions. Here we used serial sections of paraffin-embedded kidney tissues in both PAS staining and TUNEL assays. After staining, the images were captured using a Leica DM4000 microscope and TUNEL-positive cells per 0.25 mm^2^ were calculated. Apoptosis in cultured cells was analyzed by flow cytometry using Annexin V-FITC/PI apoptosis kit (70-AP101-100, MultiSciences).

### Statistics

Quantitative data were expressed as mean ± standard error of the mean. Difference was examined using two-tailed unpaired Student's t-test between two groups, or one-way ANOVA followed by Tukey's post-hoc test among three or more groups. All statistical analysis was performed using GraphPad Prism 8 software. P < 0.05 was considered statistically significant.

## Results

### TRPM2 is expressed in proximal tubules and mitochondria

**Figure 1:**
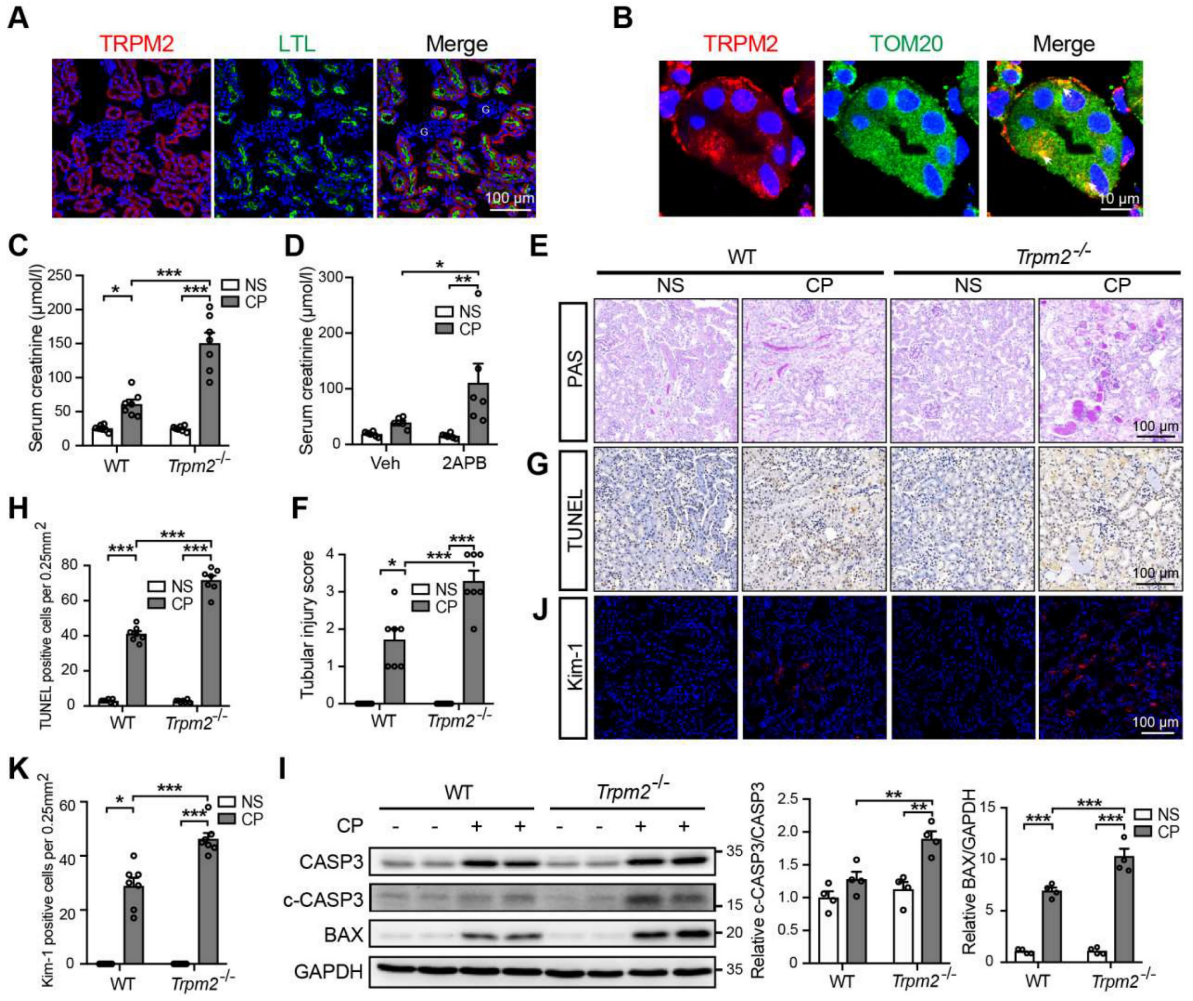
** TRPM2 deficiency aggravates cisplatin-induced acute kidney injury in mice. (A)** Representative confocal images showing TRPM2 expression in the renal cortex of WT mice without cisplatin treatment. LTL, *Lotus Tetragonolobus Lectin*; G, glomeruli. Scale bars, 100 μm. **(B)** Representative confocal images of tubular epithelial cells double-labeled TRPM2 and mitochondrial marker TOM20 (colocalization indicated by white arrows)**.** Scale bars, 10 μm. **(C)** Kidney function assessed by the serum creatinine level in *Trpm2^-/-^* and WT mice following treatment with cisplatin (CP, 18 mg/kg) or normal saline (NS) for 72 h (n = 7). **(D)** Kidney function assessed by the serum creatinine level in WT mice pretreated with vehicle or 2-APB. **(E, F)** Representative PAS staining images and quantification of tubular injury score (n = 7). Scale bars, 100 μm. **(G, H)** Representative images of TUNEL staining and quantification of apoptotic cells (n = 7). Scale bars, 100 μm. **(I)** Immunoblotting analysis and quantification of cleaved and total caspase-3 (CASP3) and BAX. **(J, K)** Representative confocal images of Kim-1 staining and quantification of Kim-1 positive cells (n = 7). Scale bars, 100 μm. Data are presented as mean ± SEM. Statistical analysis was performed using one-way ANOVA with Tukey post-hoc test. *p < 0.05, **p < 0.01, ***p < 0.001.

The absorption and accumulation of cisplatin in kidneys, particularly in renal tubular epithelial cells, leads to the nephrotoxicity 6. To examine the role of TRPM2 in cisplatin-induced kidney injury, we firstly investigated the expression of TRPM2 in kidneys. As revealed by immunofluorescent imaging, TRPM2 was expressed predominantly in the proximal tubules labelled by LTL and rarely in glomeruli or tubulointerstitium in untreated mice (Figure 1A), as previously reported 30. Similarly, TRPM2 in human kidneys had the similar distribution that was not noticeably changed due to a pathological manifestation of acute tubular necrosis Figure S1D). Typically, TRPM2 was mainly localized to the cytoplasmic membranes. Intriguingly, TRPM2 also showed diffused intracellular distribution, including in the mitochondria indicated by the co-localization of TRPM2 and the mitochondrial marker TOM20 (Figure 1B), but not in the lysosomes or endoplasmic reticulum (Figure S1E-F). Consistently, TRPM2 was detected by immunoblotting in mitochondria isolated from primary mRTECs but its mitochondrial distribution was not altered by treatment with cisplatin (Figure S1G). Finally, there seemed to be a small and transient increase in the expression of TRPM2 in kidneys after exposure to cisplatin (Figure S1H-I).

### TRPM2 deficiency aggravates cisplatin-induced AKI in mice

Next, we aimed to evaluate the role of TRPM2 in cisplatin-induced AKI. The serum creatinine level in WT mice was significantly increased by treatment with cisplatin, and such increase was aggravated by TRPM2 knockout and by addition of TRPM2 inhibitor 2-APB (Figure 1C-D). In addition, as shown by PAS staining, treatment with cisplatin resulted in apparent casts formation, necrosis of tubular epithelial cells and loss of tubular brush border in the renal cortex in WT mice, all of which became noticeably severer in *Trpm2*^-/-^ mice (Figure 1E-F). Moreover, the proportion of apoptotic cells induced by treatment with cisplatin was significantly increased in *Trpm2*^-/-^ mice compared with that in WT mice, as shown by TUNEL assay (Figure 1G-H) and immunoblotting of cleaved caspase-3 and BAX (Figure 1I). Consistently, the expression of Kim-1, a specific marker of injured tubules, shown by immunofluorescent imaging, was higher in cisplatin-treated *Trpm2*^-/-^ mice than in WT mice, indicating that TRPM2 deficiency resulted in more severe tubular injury (Figure 1J-K). Collectively, these data support that TRPM2 plays a protective role in cisplatin-induced AKI.

### TRPM2 deficiency exacerbates cisplatin-induced tubular mitochondrial injury and ROS production in kidneys

**Figure 2:**
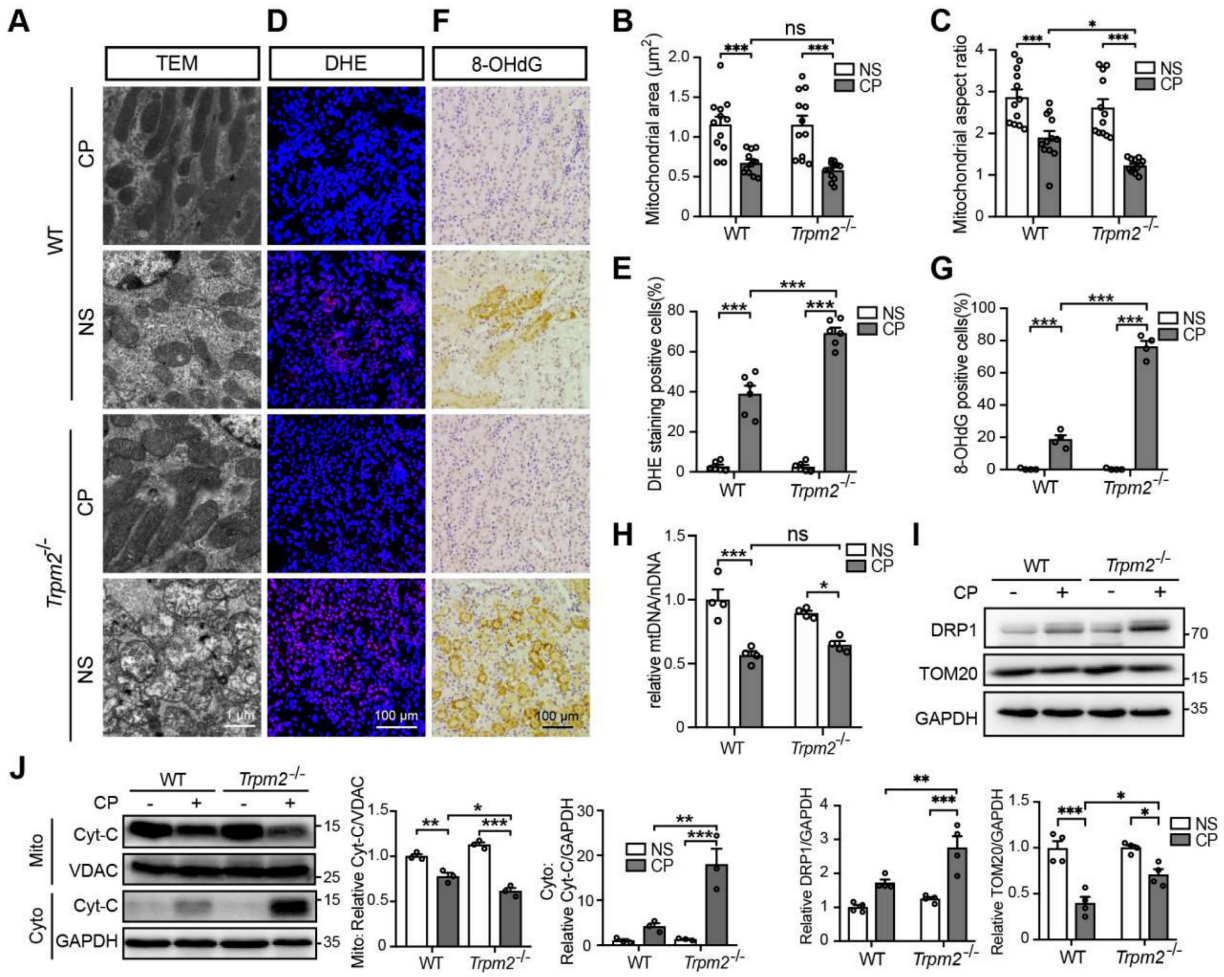
** TRPM2 deficiency exacerbates tubular mitochondrial fragmentation and oxidative damage in the kidneys of cisplatin-treated mice. (A-C)** Representative TEM images showing tubular mitochondrial morphology in the kidneys of *Trpm2^-/-^* and WT mice following treatment with cisplatin (CP, 18 mg/kg) or normal saline (NS) for 72 h and related analysis on mitochondrial area and aspect ratio. Scale bars, 1 μm.** (D, E)** Representative confocal images of DHE staining and quantification of DHE positive cells (n = 6). Scale bars, 100 μm. **(F, G)** Representative immunohistochemistry images of 8-OHdG staining and quantification of 8-OHdG positive cells (n = 6). Scale bars, 100 μm. **(H)** Relative mitochondrial DNA content calculated by the ratio of mitochondrial DNA (mtDNA) to nuclear DNA (nDNA) (n = 4).** (I)** Immunoblotting analysis and quantification of DRP1 and TOM20 in kidneys. **(J)** Immunoblotting analysis and quantification of cytochrome-c (Cyt-c) in the fractions of mitochondria and cytoplasm deprived of mitochondria. Cyto, cytoplasm; Mito, mitochondria. Data are presented as mean ± SEM. Statistical analysis was performed using one-way ANOVA with Tukey post-hoc test. *p < 0.05, **p < 0.01, ***p < 0.001.

Mitochondrial dysfunction is known to be closely associated with cisplatin-induced tubular cell death and kidney injury. TRPM2 in cardiomyocytes was reported to be indispensable for maintaining mitochondrial function (26. Moreover, genetic depletion of TRPM2 in neuroblastoma impaired the expression and activation of mitochondrial proteins through downregulating cAMP-responsive element-binding protein and proline-rich tyrosine kinase 2 40. Hence, we sought to investigate the role of TRPM2 on the integrity and function of mitochondria in kidneys. As shown using TEM imaging, treatment with cisplatin led to mitochondrial damage in the tubular cells of renal cortex, which was characterized by brightened matrix, mitochondrial swelling, loss of cristae and even mitochondrial rupture (Figure 2A). These alterations in the renal tubules were substantially greater, albeit no spontaneous mitochondrial abnormalities in morphology, in *Trpm2*^-/-^ mice (Figure 2A). Morphology analysis revealed significant declines in mitochondrial area and aspect ratio after exposure to cisplatin, with the latter being more apparent in *Trpm2*^-/-^ kidneys (Figure 2B-C). Treatment with cisplatin markedly increased DHE-positive cells, indicating an elevated level of ROS in kidneys, and the increase was significantly higher in cells from *Trpm2*^-/-^ mice (Figure 2D-E). In line with these observations, treatment with cisplatin induced a higher level of oxidative damage to DNA, demonstrated by 8-OHdG staining in *Trpm2*^-/-^ mice than in WT mice (Figure 2F-G). There was also a decline in the mtDNA level after treatment with cisplatin, which was however not significantly different between WT and *Trpm2*^-/-^ mice (Figure 2H). Meanwhile, cisplatin led to upregulation of mitochondrial fission marker DRP1 which was exacerbated by TRPM2 deficiency and downregulation of mitochondrial membrane marker TOM20 which was inhibited by TRPM2 deficiency (Figure 2I). Moreover, treatment with cisplatin induced release of Cyt-c into the cytoplasm in kidneys, shown by immunoblotting, which was augmented in *Trpm2*^-/-^ mice (Figure 2J). Taken together, these data suggest that TRPM2 is critically engaged in maintaining tubular mitochondrial function in response to treatment with cisplatin.

### Inhibition of TRPM2 worsens cisplatin-induced cell apoptosis and mitochondrial dysfunction *in vitro*

**Figure 3:**
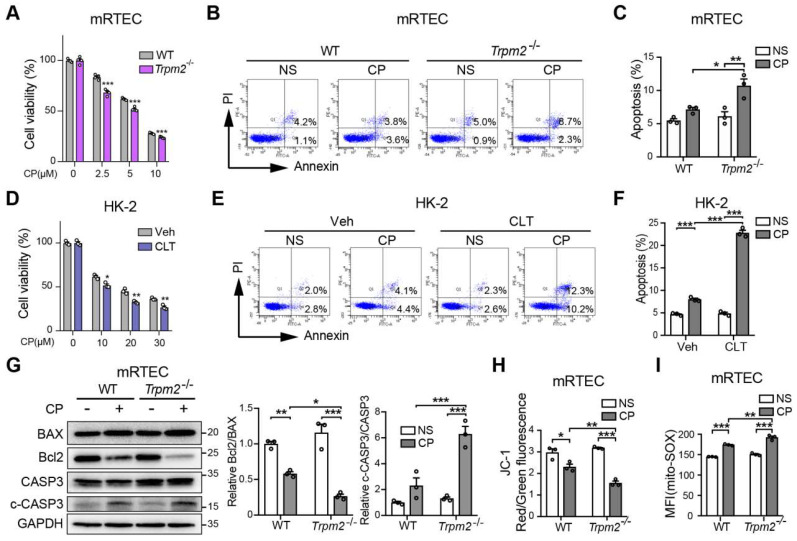
** TRPM2 deficiency increases cisplatin-induced cell apoptosis and mitochondrial dysfunction in tubular cells. (A)** Comparison of cell viability between* Trpm2*^-/-^ and WT mRTECs exposed to indicated concentrations of cisplatin (CP) for 24 h (n = 4). **(B, C)** Cell apoptosis determined by flow cytometry in mRTECs treated with 5 μM CP or normal saline (NS) for 24 h (n = 3). **(D)** Cell viability of HK-2 cells incubated with TRPM2 inhibitor clotrimazole (CLT, 20 μM) or vehicle (Veh) for 1 h and then subjected to cisplatin (CP) at indicated concentrations for 24 h (n = 3). **(E, F)** Cell apoptosis determined by flow cytometry in HK-2 cells treated with 20 μM CP or normal saline (NS) for 24 h (n = 3). **(G)** Immunoblotting analysis and quantification of BAX, Bcl2, cleaved and total caspase-3 (CASP3) in mRTECs. **(H)** Mitochondrial membrane potential in mRTECs indicated by the ratio of red to green fluorescence intensity of JC-1 (n = 3).** (I)** Level of mitochondrial ROS in mRTECs determined by the mean fluorescence intensity (MFI) of Mito-SOX (n = 3). Data are presented as mean±SEM. Statistical analysis was performed using two-tailed unpaired Student's t-test in** (A, D)** and one-way ANOVA with Tukey post-hoc test in** (C, F, G, H and I)**. *p < 0.05, **p < 0.01, ***p < 0.001.

We further explored the role of TRPM2 in cisplatin-triggered cell death *in vitro*. Treatment with cisplatin concentration-dependently reduced the viability of primary mRTECs and MEFs isolated from mice evaluated by the cell counting kit-8 (CCK-8) assay (Figure 3A and S2A), and induced apoptotic cell death determined by flow cytometry (Figure 3B-C and S2B). Cisplatin-induced cytotoxicity and apoptosis was worsened by TRPM2 deficiency. Similarly, cisplatin-induced cytotoxicity and apoptosis was sensitized by treatment with CLT, a blocker of TRPM2 channel in human proximal tubular epithelial cell line HK-2 cells (Figure 3D-F). Treatment with cisplatin induced an increase in cleaved caspase-3 and a decrease in the ratio of Bcl2 to BAX, demonstrated by immunoblotting, both of which became more prominent in mRTECs (Figure 3G) and MEFs Figure S2C) from *Trpm2^-/-^* mice. These data provide further evidence to reinforce the protective role of TRPM2 against cisplatin-induced toxicity to renal tubular cells.

We also assessed the effects of treatment with cisplatin on mitochondrial function. In both mRTECs and MEFs, treatment with cisplatin reduced the ratio of red to green fluorescence intensity of JC-1 (Figure 3H and S2D), suggesting loss of MMP, which was exacerbated by TRPM2 deficiency. On the contrary, treatment with cisplatin markedly increased the fluorescence intensity of MitoSOX, an indicator of mitochondrial superoxide, in mRTECs and MEFs from WT mice and to a higher level in cells from *Trpm2^-/-^* mice (Figure 3I and S2E). Treatment with cisplatin elicited mitochondrial fission, detected by Mitotracker Red, which was also aggravated by TRPM2 deficiency (Figure S2F). Of notice, TRPM2 deficiency alone did not affect MMP, mtROS level and mitochondrial morphology, suggesting that TRPM2 causes no mitochondrial damage in the absence of stress. Collectively, the results support that TRPM2 plays a crucial role in protecting against cisplatin-induced cell apoptosis and mitochondrial dysfunction.

### Mito-TEMPO rescues increased tubular mitochondrial damage and cell apoptosis in *Trpm2^-/-^* mice during cisplatin-induced kidney injury

**Figure 4:**
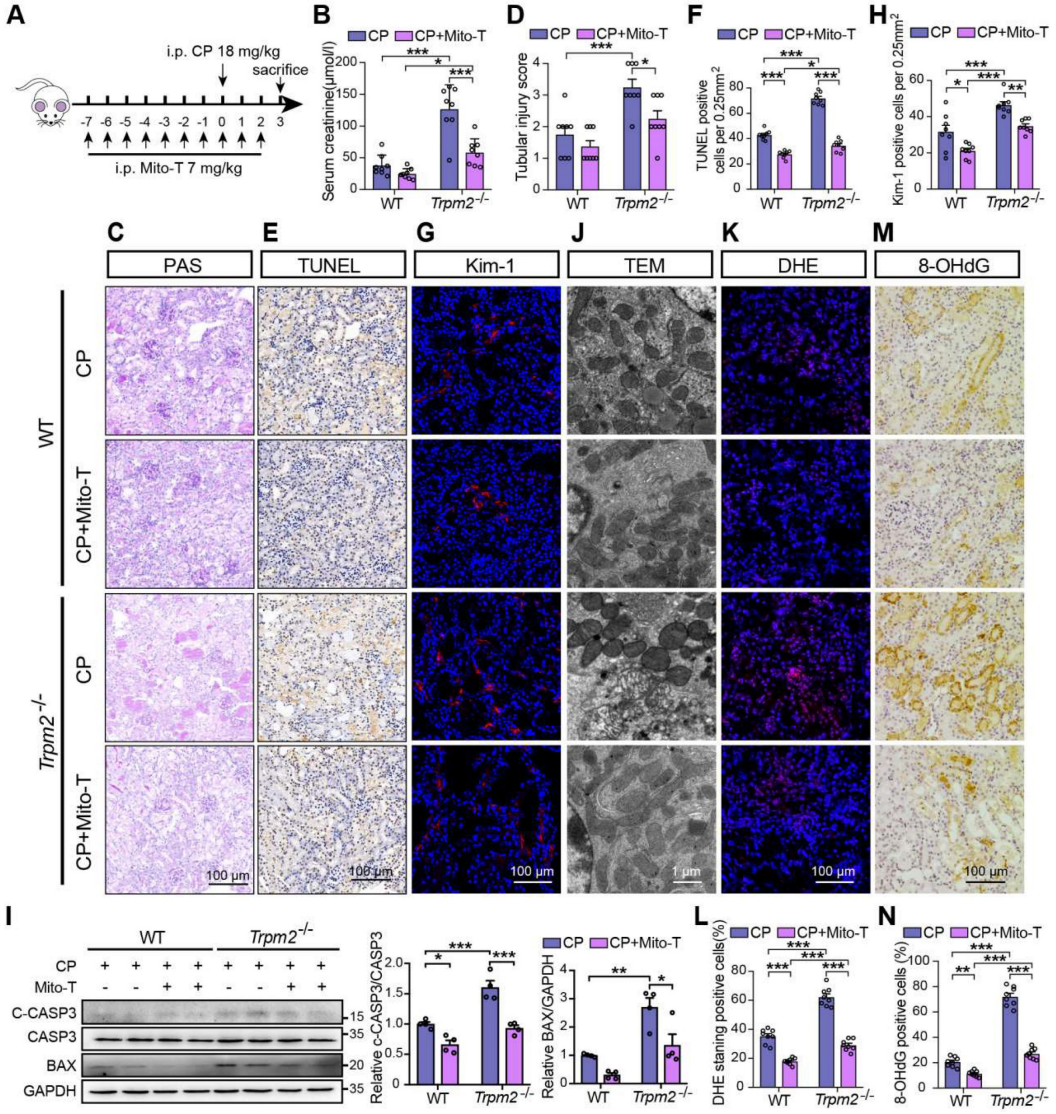
** Mitochondrial ROS scavenger Mito-TEMPO protects mice from cisplatin-induced kidney injury and mitochondrial damage. (A)** Schematic diagram of the experimental design**.** Briefly, *Trpm2^-/-^* and WT mice were injected daily with either Mito-TEMPO (Mito-T, 7 mg/kg) or vehicle one week before administration of cisplatin (CP, 18 mg/kg) until being sacrificed at 3 days. **(B)** Kidney function assessed by the serum creatinine level (n = 8). **(C, D)** Representative PAS staining images and quantification of tubular injury score (n = 8). Scale bars, 100 μm.** (E, F)** Representative images of TUNEL staining and quantification of apoptotic cells (n = 8). Scale bars, 100 μm. **(G, H)** Representative confocal images of Kim-1 staining and quantification of Kim-1 positive cells (n = 8). Scale bars, 100 μm. **(I)** Immunoblotting analysis and quantification of BAX and cleaved and total caspase-3 (CASP3) in mice**. (J)** Representative TEM images showing mitochondrial morphology in the renal tubules. Scale bars, 1 μm.** (K, L)** Representative confocal images of DHE staining and quantification of DHE positive cells (n = 8). Scale bars, 100 μm. **(M, N)** Representative immunohistochemistry images of 8-OHdG staining and quantification of 8-OHdG positive cells (n = 8)**.** Scale bars, 100 μm. Data are presented as mean±SEM. Statistical analysis was performed using one-way ANOVA with Tukey post-hoc test. *p < 0.05, **p < 0.01, ***p < 0.001.

Based on the findings described above, we hypothesized that increased mitochondrial dysfunction and mtROS production were responsible for the deterioration of kidney injury due to TRPM2 silencing. Mito-TEMPO was shown to be effective in reducing ischemic AKI (41 and renal fibrosis 42 through eliminating excessive mtROS. Therefore, we tested this hypothesis by determining the effects of pretreatment with Mito-TEMPO (Figure 4A). As expected, treatment with Mito-TEMPO clearly ameliorated renal dysfunction (Figure 4B) and tubular injury (Figure 4C-D) in both WT and *Trpm2^-/-^* mice, but the WT mice experienced mild even insignificant improvement on renal function and tubular injury (Figure 4B-D). Treatment with Mito-TEMPO also effectively reduced the number of apoptotic cells (Figure 4E-F) and Kim-1 positive tubular cells (Figure 4G-H) and the expression of apoptotic biomarker (Figure 4I) in both cisplatin-treated WT and *Trpm2^-/-^* mice. In addition, the fragmentation of tubular mitochondria was considerably lessened in cisplatin-treated *Trpm2^-/-^* mice following administration with Mito-TEMPO (Figure 4J). Moreover, the proportions of DHE-positive cells and 8-OHdG-positive cells were substantially decreased in *Trpm2^-/-^* mice compared that in WT mice (Figure 4K-N). In summary, elimination of mtROS by Mito-TEMPO preserved the integrity of mitochondria, reduced oxidative damage and alleviated cell apoptosis and renal dysfunction in cisplatin-treated mice, particularly in *Trpm2^-/-^* mice. These data support the notion that TRPM2 activation mediates the protection against cisplatin-induced mitochondrial dysfunction, apoptosis and loss of renal function, likely through reducing mtROS production.

### TRPM2 is required for cisplatin-induced activation of autophagy

**Figure 5:**
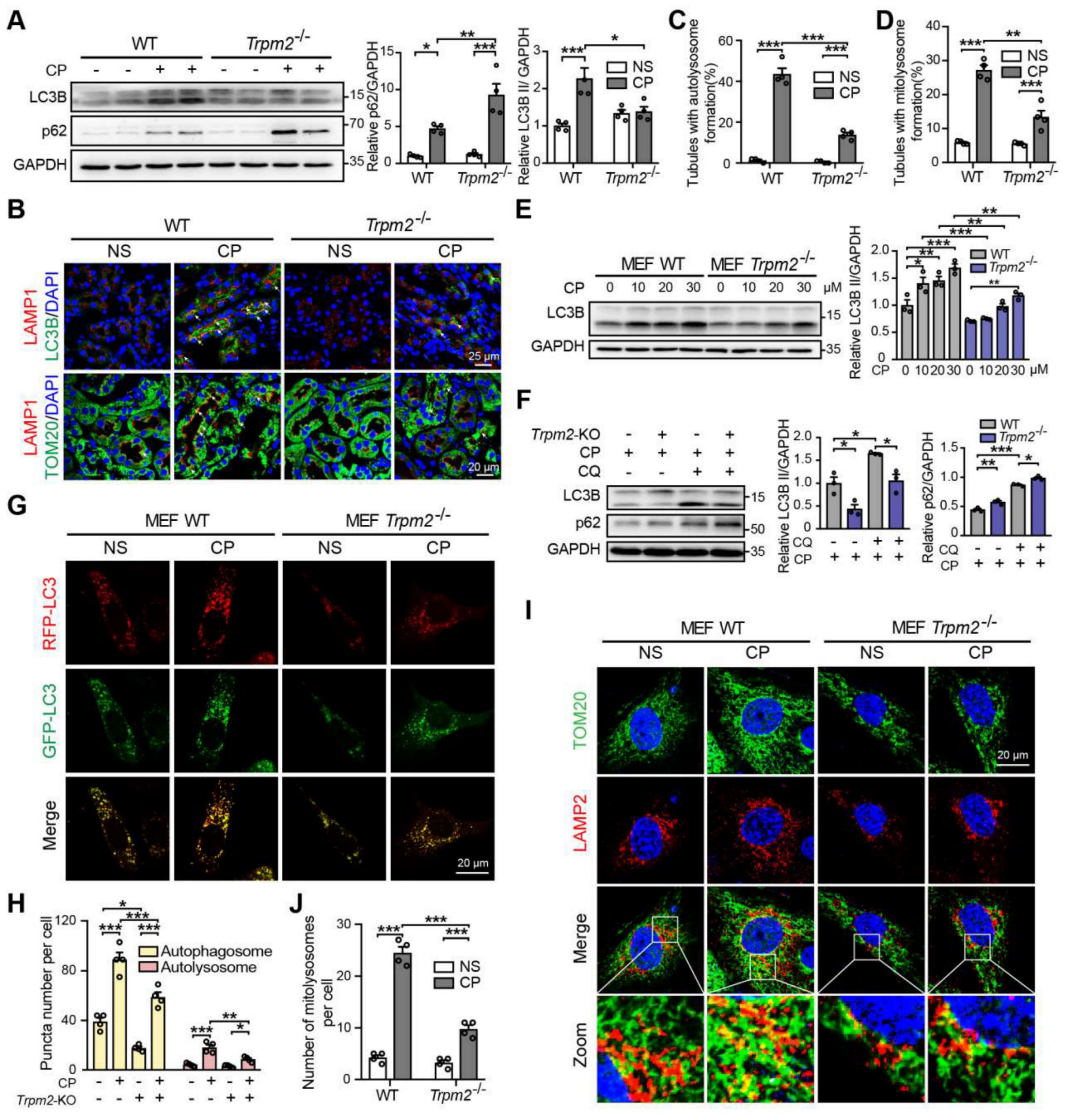
** TRPM2 is required for cisplatin-evoked autophagy *in vitro* and *in vivo*. (A)** Immunoblotting analysis and quantification of LC3B II and p62 in the kidneys of *Trpm2^-/-^* and WT mice following treatment with cisplatin (CP, 18 mg/kg) or normal saline (NS) for 72 h.** (B)** Formation of autolysosomes in the kidneys evaluated by immunofluorescence double-labeled LC3B and LAMP1. Scale bars, 25 μm. Formation of mitolysosomes in the kidneys evaluated by immunofluorescence double-labeled TOM20 and LAMP1. Scale bars, 20 μm. Quantification of **(C)** autolysosomes and **(D)** mitolysosomes in the renal tubules (n = 4). **(E)** Immunoblotting analysis and quantification of LC3B II in *Trpm2^-/-^* and WT MEFs treated with the indicated concentrations of CP for 24 h. **(F)** Immunoblotting analysis and quantification of LC3B II and p62 in *Trpm2^-/-^* and WT MEFs incubated with 10 μM chloroquine (CQ) or vehicle for 1 h before treatment with 20 μM CP for 24 h. **(G)** Representative confocal images of LC3 puncta in *Trpm2^-/-^* and WT MEFs expressing *mRFP-GFP-LC3.* Scale bars, 20 μm. **(H)** Quantification of autophagosomes (yellow dots, RFP^+^GFP^+^) and autolysosomes (red dots, RFP^+^GFP^-^) (n = 4). **(I)** Formation of mitolysosomes in *Trpm2^-/-^* and WT MEFs evaluated by immunofluorescence double-labeled TOM20 and LAMP2. Scale bars, 20 μm. **(J)** Quantification of mitolysosomes in MEFs (n = 4). Data are presented as mean±SEM. Statistical analysis was performed using one-way ANOVA with Tukey post-hoc test. *p < 0.05, **p < 0.01, ***p < 0.001.

Autophagy is one of the most important mechanisms for degrading damaged organelles, including mitochondria. Increasing evidence from recent studies suggests strong connection of TRPM2 with induction of autophagy 27-29, 43. We were interested in whether autophagy, particularly TRPM2-mediated autophagy is engaged in cisplatin-induced AKI. Treatment with cisplatin resulted in an increase in autophagic marker LC3B II in mouse kidneys, revealed by immunoblotting analysis, which was attenuated by TRPM2 deficiency (Figure 5A). Treatment with cisplatin led to accumulation of the selective autophagy receptor p62, which was higher in *Trpm2^-/-^* mice, indicating compromised autophagic clearance (Figure 5A). Autolysosomes form by the fusion of autophagosomes and lysosomes for the later substrate degradation. Treatment with cisplatin induced co-localization of LC3 puncta and lysosomal marker LAMP1 in renal cortex, suggesting formation of autolysosomes, which was substantially reduced in *Trpm2^-/-^* mice (Figure 5B-C). Mitophagy is a selective form of autophagy for degrading damaged mitochondria 1. We further evaluated the formation of mitolysosomes, a late stage of mitophagy. The number of mitolysosomes in renal cortex, shown by co-localization of TOM20 and LAMP1, was increased after treatment with cisplatin, and such cisplatin-induced formation of mitolysosomes was inhibited by TRPM2 deficiency (Figure 5B, D).

In line with the above *in vivo* findings, treatment with cisplatin increased the expression of LC3B II in a concentration-dependent manner in MEFs from WT mice, but this effect was largely blunted in MEFs from *Trpm2^-/-^* mice (Figure 5E). The accumulation of LC3B II and p62 was elevated further in MEFs treated with CQ, an inhibitor of lysosomal function. In the presence of CQ, the expression of LC3B II was also downregulated in TRPM2-deificent MEFs, indicating suppressed autophagic flux (Figure 5F). In addition, the formation of autophagosomes and autolysosomes was further assessed in cells that expressed mRFP-GFP-LC3 (Figure 5G). Consistently, the increased levels of both autophagosomes and autolysosomes after treatment with cisplatin were downregulated by TRPM2 deficiency (Figure 5H). Moreover, treatment with cisplatin led to a notable increase in the number of mitolysosomes, i.e. co-localization of TOM20 and LAMP2, which was markedly reduced by TRPM2 silencing (Figure 5I-J), indicating a crucial role for TRPM2 in eliminating damaged mitochondria. Taken together, these data suggest that TRPM2 is required for cisplatin-induced autophagy *in vitro* and *in vivo.*

### TRPM2 protects against cisplatin-induced kidney injury and mitochondrial damage by modulation of autophagy through inhibiting the AKT-mTOR pathway

**Figure 6:**
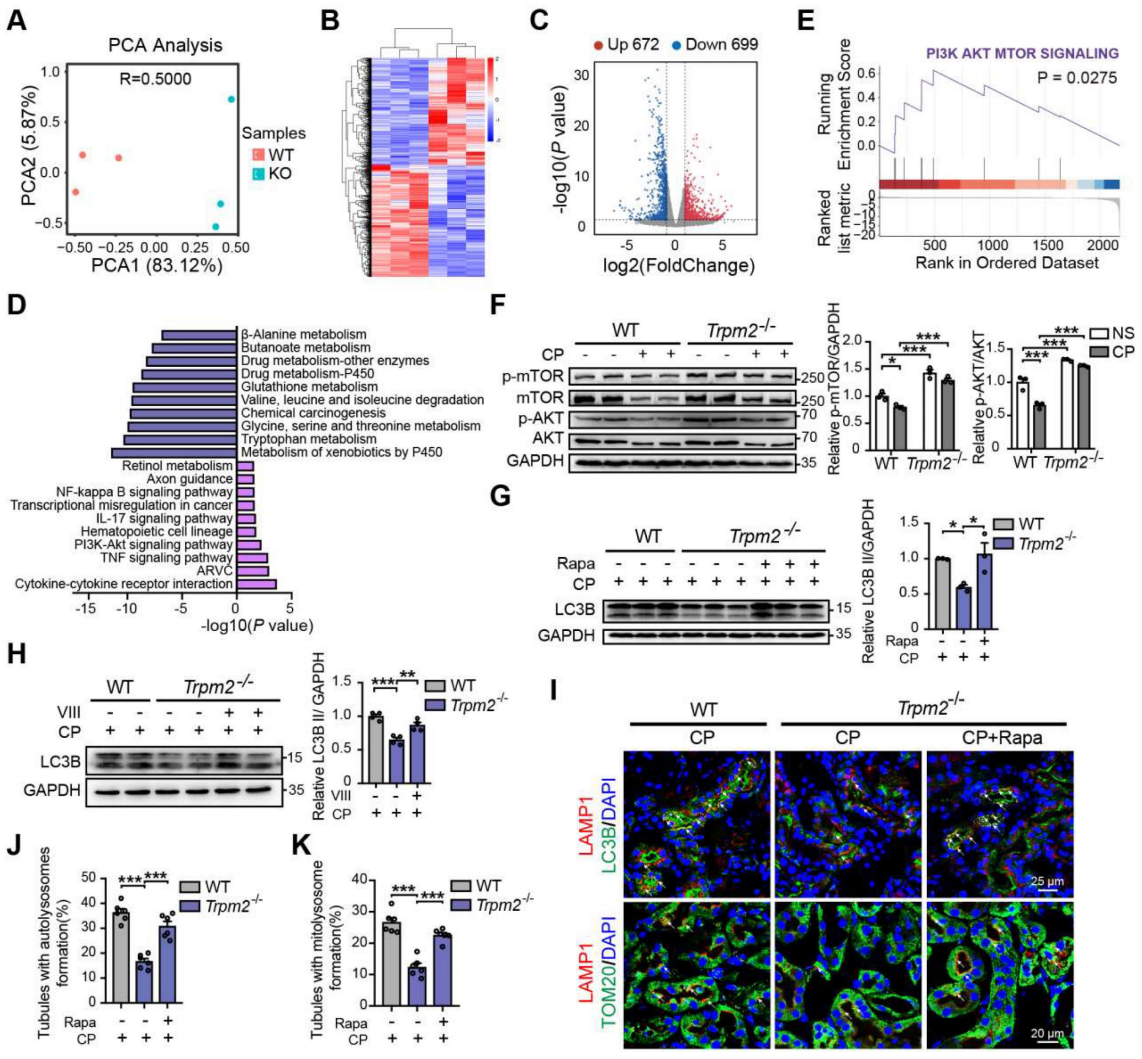
** TRPM2 regulates cisplatin-induced autophagy in kidneys via suppressing the AKT-mTOR signaling pathway. (A)** PCA analysis on the RNA-sequencing data from cisplatin (CP)-treated* Trpm2^-/-^* and WT mice (n = 3). **(B, C)** Heatmap and volcano plot of differentially expressed genes (|log2(FoldChange)| > 1, *P* value < 0.05). **(D)** Top 10 upregulated and downregulated Kyoto Encyclopedia of Genes and Genomes pathway enrichments. ARVC, Arrhythmogenic Right Ventricular Cardiomyopathy; TNF, tumor necrosis factor.** (E)** Gene Set Enrichment Analysis of PI3K-AKT-mTOR pathway generated from *Trpm2^-/-^* and WT mice subjected to cisplatin. **(F)** Immunoblotting analysis and quantification of p-mTOR, mTOR, p-AKT, AKT in the kidneys of *Trpm2^-/-^* and WT mice. **(G)** Immunoblotting analysis and quantification of LC3B II in the kidneys of CP-treated mice. The mice were either injected i.p. with 1 mg/kg rapamycin (Rapa) or vehicle 1 hour prior to and 1 day after CP treatment. **(H)** Immunoblotting analysis and quantification of LC3B II in the kidneys of CP-treated mice. The mice were injected i.p. with either 50 mg/kg VIII or vehicle 1 h prior to cisplatin injection. **(I)** Formation of autolysosomes evaluated by immunofluorescence double-labeled LC3B and LAMP1. Scale bars, 25 μm. Formation of mitolysosomes evaluated by immunofluorescence double-labeled TOM20 and LAMP1. Scale bars, 20 μm. Quantification of **(J)** autolysosomes and **(K)** mitolysosomes in the renal tubules (n = 6). Data are presented as mean±SEM. Statistical analysis was performed using one-way ANOVA with Tukey post-hoc test. *p < 0.05, **p < 0.01, ***p < 0.001.

To elucidate the mechanism underlying the association between TRPM2 and cisplatin-induced kidney injury, we performed kidney RNA-sequencing analysis. Distinct gene expression profile between WT and *Trpm2^-/-^* kidneys was initially illustrated by PCA analysis (Figure 6A). A total of 672 genes were significantly increased and 699 genes were significantly decreased in *Trpm2^-/-^* mice (Figure 6B-C). The top downregulated pathways revealed by KEGG analysis were enriched in several metabolism pathways, whereas the top upregulated pathways were mainly enriched in inflammatory responses including TNF, IL-17 and NF-κB signaling pathways and cell survival-related PI3K-AKT pathways (Figure 6D). It is worth noting that the AKT-mTOR signaling pathway is well known for autophagy inhibition 44-46 and is enriched by GSEA analysis in this study (Figure 6E). Here, the phosphorylation levels of AKT and downstream mTOR were both significantly increased in the kidneys of *Trpm2^-/-^* mice compared with those in WT mice (Figure 6F). We subsequently used rapamycin and VIII to block the activity of mTOR and AKT, respectively, in cisplatin-treated *Trpm2^-/-^* mice. Treatment with rapamycin and VIII both markedly elevated the LC3B II level in *Trpm2^-/-^* mice by immunoblotting analysis (Figure 6G-H). In addition, rapamycin markedly increased the number of autolysosomes (Figure 6I-J) and mitolysosomes (Figure 6I, K) in renal tubules of *Trpm2^-/-^* mice to a level that was approximate to that in WT mice. These results suggest that the AKT-mTOR signaling pathway is critically involved in TRPM2-mediated autophagy activation *in vivo*.

**Figure 7:**
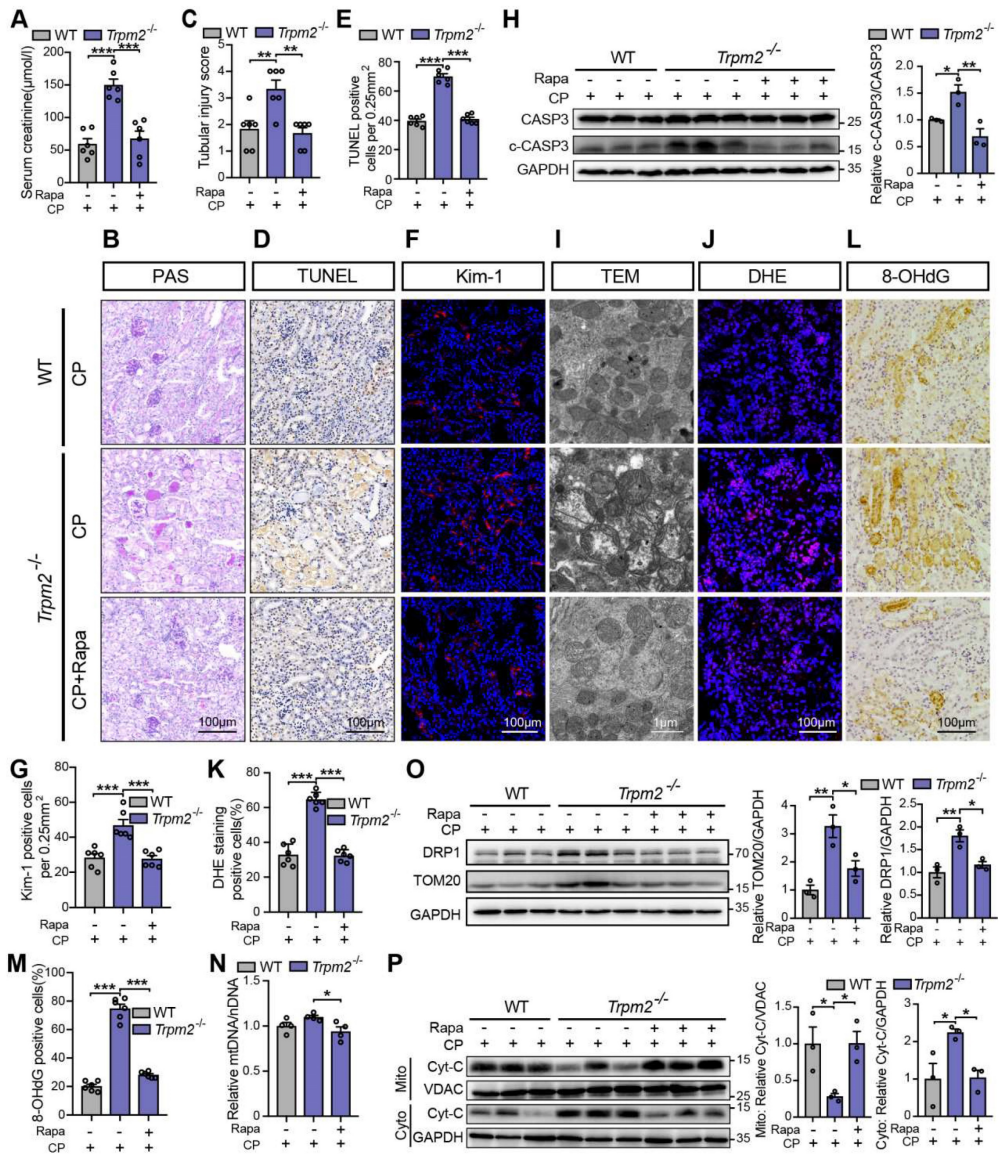
** mTOR inhibitor rapamycin alleviates cisplatin-induced kidney injury, cell apoptosis and mitochondrial dysfunction in *Trpm2^-/-^* mice. (A)** Kidney function assessed by serum creatinine level in cisplatin (CP, 18 mg/kg)-treated *Trpm2^-/-^* and WT mice (n = 6). The mice were either injected i.p. with 1 mg/kg rapamycin (Rapa) or vehicle 1 hour prior to and 1 day after CP treatment.** (B, C)** Representative PAS staining images and quantification of tubular injury score (n = 6). Scale bars, 100 μm.** (D, E)** Representative images of TUNEL staining and quantification of apoptotic cells (n = 6). Scale bars, 100 μm. **(F, G)** Representative confocal images of Kim-1 staining and quantification of Kim-1 positive cells (n = 6). Scale bars, 100 μm. **(H)** Immunoblotting analysis and quantification of cleaved and total caspase-3 (CASP3).** (I)** Representative TEM images showing the mitochondrial morphology in the renal tubules. Scale bars, 1 μm.** (J, K)** Representative confocal images of DHE staining and quantification of DHE positive cells (n = 6). Scale bars, 100 μm. **(L, M)** Representative immunohistochemistry images of 8-OHdG staining and quantification of 8-OHdG positive cells (n = 6)**.** Scale bars, 100 μm.** (N)** Relative mitochondrial DNA content calculated by the ratio of mitochondrial DNA (mtDNA) to nuclear DNA (nDNA) (n = 4). **(O)** Immunoblotting analysis and quantification of DRP1 and TOM20. **(P)** Immunoblotting analysis and quantification of cytochrome-c (Cyt-c) in the fractions of mitochondria and cytoplasm deprived of mitochondria. Cyto, cytoplasm; Mito, mitochondria. Data are presented as mean±SEM. Statistical analysis was performed using one-way ANOVA with Tukey post-hoc test. *p < 0.05, **p < 0.01, ***p < 0.001.

**Figure 8:**
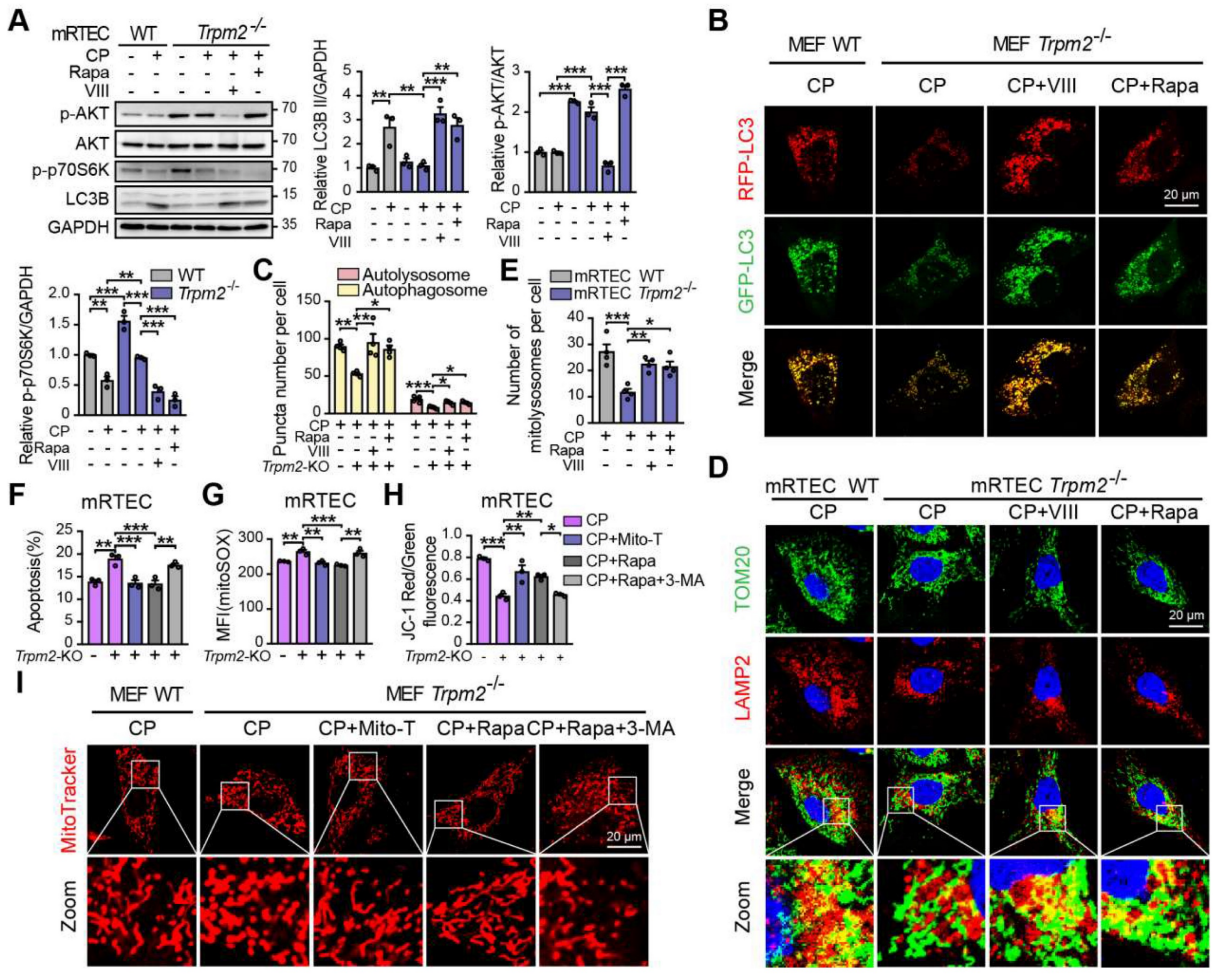
** TRPM2 protects against cisplatin-induced cell apoptosis and mitochondrial dysfunction *in vitro* by the regulation of autophagy via suppressing the AKT-mTOR signaling pathway.** *Trpm2^-/-^* MEFs or mRTECs were pre-incubated with AKT inhibitor VIII (5 μM) or mTOR inhibitor rapamycin (Rapa, 50 nM) for 1 h before cisplatin (CP) intervention. **(A)** Immunoblotting analysis and quantification of LC3B II, p-AKT and p-p70S6K in *Trpm2^-/-^* and WT mRTECs following treatment with 5 μM CP for 24 h. **(B)** Representative confocal images of LC3 puncta in* Trpm2^-/-^* and WT MEFs expressing *mRFP-GFP-LC3 f*ollowing treatment with 20 μM CP for 24 h*.* Scale bars, 20 μm. **(C)** Quantification of autophagosomes (yellow dots, RFP^+^GFP^+^) and autolysosomes (red dots, RFP^+^GFP^-^) (n = 4).** (D, E)** Representative confocal images and quantification of mitolysosomes evaluated by immunofluorescence double-labeled TOM20 and LAMP2 in mRTECs (n = 4). Scale bars, 20 μm. Then, *Trpm2^-/-^* mRTECs and MEFs were pre-incubated with rapamycin (50 nM), Mito-TEMPO (Mito-T, 200 nM) or Rapamycin (50 nM) plus 3-MA (5 mM) for 1 h followed by intervention of 5 and 20 μM CP for 24 h, respectively. **(F)** CP-induced apoptosis in mRTECs determined by flow cytometry (n = 3). **(G)** Level of mitochondrial ROS in mRTECs determined by the mean fluorescent intensity (MFI) of Mito-SOX (n = 3).** (H)** Mitochondrial membrane potential of mRTECs indicated by the ratio of red to green fluorescence intensity of JC-1 (n = 3). **(I)** Mitochondrial morphology of MEFs detected by the fluorescence of MitoTracker Red. Scale bars, 20 μm. Data are presented as mean±SEM. Statistical analysis was performed using one-way ANOVA with Tukey post-hoc test. *p < 0.05, **p < 0.01, ***p < 0.001.

We subsequently examined the role of TRPM2-mediated autophagy in cisplatin-induced tubular mitochondrial dysfunction and kidney injury. As expected, treatment with rapamycin resulted in a marked decline of the serum creatinine level in *Trpm2^-/-^* mice (Figure 7A), accompanied by reduced degree of tubular damage (Figure 7B-C) and number of TUNEL positive cells (Figure 7D-E) and Kim-1 positive cells in the renal cortex (Figure 7F-G). The alterations in the expression of cleaved caspase-3 (Figure 7H) confirmed an evident amelioration of apoptosis in kidneys following treatment with rapamycin. Furthermore, mitochondrial damage was lessened in the renal tubules (Figure 7I), consistent with reduced oxidative damage, indicated by reduced DHE positive (Figure 7J-K) and 8-OHdG positive (Figure 7L-M) cells. The content of mtDNA was further decreased after treatment with rapamycin, indicating strengthened elimination of damaged mitochondria (Figure 7N). Consistently, the upregulation of DRP1 and TOM20 in *Trpm2^-/-^* mice was evidently inhibited by the addition of rapamycin (Figure 7O). Furthermore, treatment with rapamycin increased localization of Cyt-c in the mitochondria and reduced its release to the cytosol, suggesting improved mitochondrial integrity (Figure 7P). Collectively, these data suggest that TRPM2-mediated inhibition of mTOR plays a key role in the regulation of autophagy and the alleviation of mitochondrial damage and cell apoptosis associated with cisplatin-induced AKI.

### TRPM2 protects against cisplatin-induced cell apoptosis and mitochondrial damage *in vitro* by modulation of autophagy through inhibiting the AKT-mTOR pathway

We also investigated the role of the AKT-mTOR signaling pathway in cisplatin-treated cells. The phosphorylation level p70S6K, downstream of mTOR is considered a marker of the mTOR activity 47. Treatment with cisplatin reduced the phosphorylation levels of AKT and p70S6K, both of which being higher in TRPM2-deficent mRTECs (Figure 8A) and MEFs Figure S3A). Administration of rapamycin or AKT inhibitor VIII dramatically inhibited the phosphorylation level of p70S6K and increased the expression of LC3B II in TRPM2 deficient cells (Figure 8A and S3A). Further, autophagic flux was suppressed due to TRPM2 silencing as shown by the declined number of autophagosome and autolysosome, both of which were restored by treatment with rapamycin and VIII (Figure 8B-C). Consistently, the reduced number of mitolysosomes in TRPM2 deficient mRTECs and MEFs were also substantially increased by treatment with rapamycin and VIII (Figure 8D-E and S3B-C). These data further demonstrate that TRPM2 mediates cisplatin-induced autophagy via modulating the AKT-mTOR signaling pathway.

Next, we examined the effects of TRPM2-mediated autophagy on mitochondrial homeostasis and cell apoptosis. Treatment with rapamycin not only improved the autophagic level but also led to a marked increase in cell viability and a marked decrease in apoptotic cells in TRPM2 deficient mRTECs and MEFs, similar with the effects of Mito-TEMPO (Figure 8F and S3D-E). However, concomitant use of 3-MA, a widely used inhibitor of autophagy, blocked the anti-apoptotic effects of rapamycin (Figure 8F and S3E). Furthermore, like treatment with Mito-TEMPO, treatment with rapamycin effectively attenuated the increase of mtROS production (Figure 8G and S3F), and rescued the decline of MMP (Figure 8H and S3G) and the fragmentation of mitochondria caused by cisplatin (Figure 8I). However, all of these beneficial effects were blocked by treatment with 3-MA. These data indicate that cisplatin-caused mitochondrial damage and cell apoptosis are worsened by TRPM2 deficiency through perturbation of the AKT-mTOR pathway-mediated autophagy.

### TRPM2-mediated Ca^2+^ influx modulates the AKT-mTOR signaling pathway

**Figure 9:**
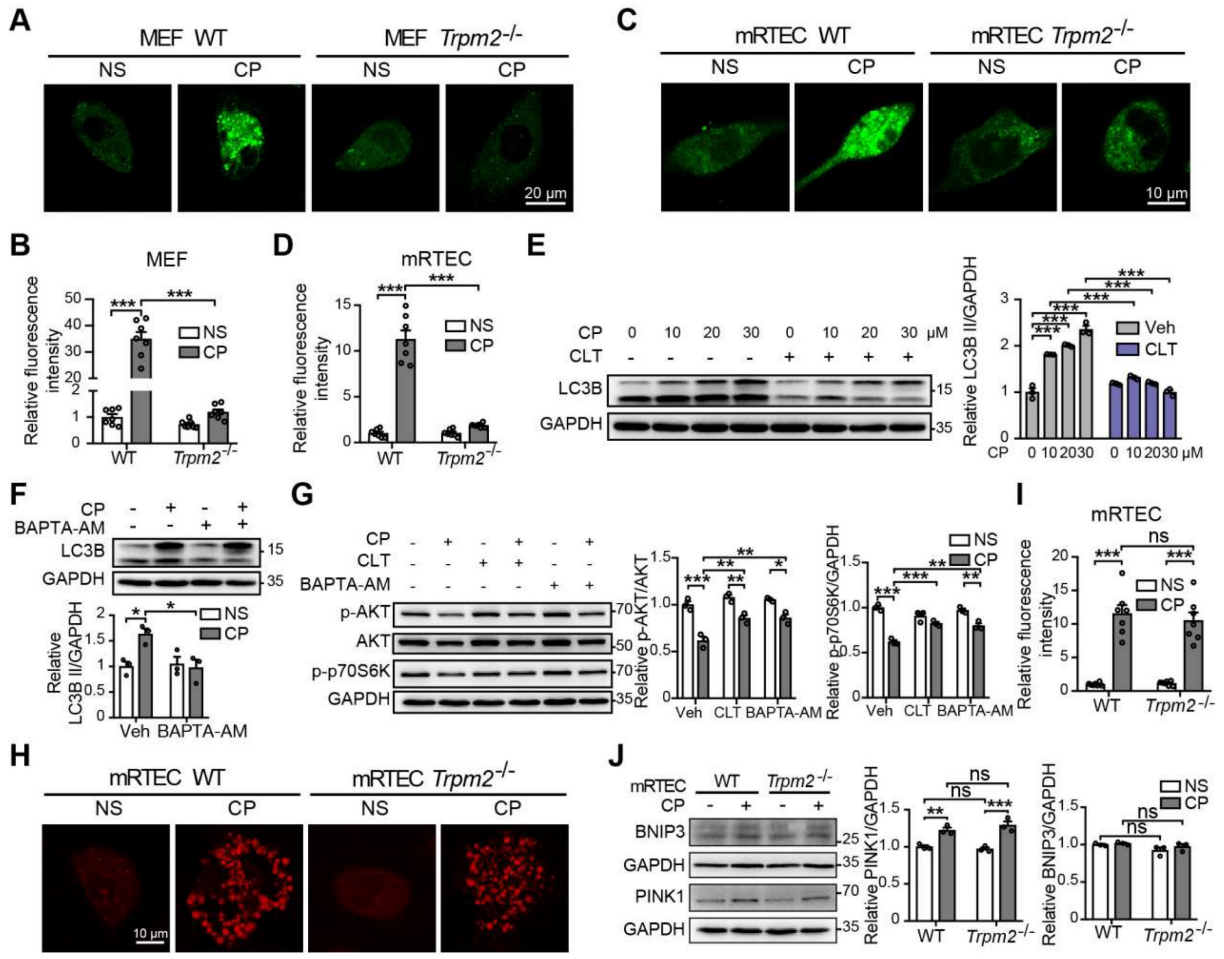
** TRPM2-mediated Ca^2+^ influx is involved in the inhibition of the AKT-mTOR pathway in response to cisplatin.** Intracellular Ca^2+^ signals indicated by the fluorescence of Fluo-4 AM in cisplatin (CP, 20 μM) or normal saline (NS) treated *Trpm2^-/-^* and WT MEFs **(A, B)** and mRTECs** (C, D)** (n = 7). Scale bars, 20 μm and 10μm, respectively. **(E)** Immunoblotting analysis and quantification of LC3B II in WT MEFs after incubation with clotrimazole (CLT, 10 μM) or vehicle (Veh) for 1 h followed by treatment with CP at the indicated concentration for 24 h. **(F)** Immunoblotting analysis and quantification of LC3B II in WT MEFs after incubation with 5 μM BAPTA-AM for 1 h followed by treatment with 20 μM CP for 24 h. **(G)** Immunoblotting analysis and quantification of p-AKT, AKT and p-p70S6K in WT MEFs after incubation with 5 μM BAPTA-AM or 10 μM clotrimazole for 1 h followed by treatment with 20 μM CP for 24 h. **(H, I)** Mitochondrial Ca^2+^ signals indicated by the fluorescence of Rhod-2 in mRTECs treated by CP or NS (n = 7). Scale bars, 10 μm. **(J)** Immunoblotting analysis and quantification of BNIP3 and PINK1 in mRTECs treated by CP or NS. Data are presented as mean±SEM. Statistical analysis was performed using one-way ANOVA with Tukey post-hoc test. ns, not significant; *p < 0.05, **p < 0.01, ***p < 0.001.

Accumulating studies show that Ca^2+^ plays multifaceted roles in the regulation of autophagy (48, raising the question of whether TRPM2-mediated Ca^2+^ influx facilitates autophagy induction and, if it does, whether this effect depends on the AKT-mTOR signaling pathway. We firstly evaluated the intracellular Ca^2+^ level in MEFs and mRTECs using Fluo-4 AM, a fluorescent Ca^2+^ indicator. Exposure to cisplatin induced a significant increase in the fluorescence intensity, indicating an increase in intracellular Ca^2+^ level, which was ablated by TRPM2 deficiency (Figure 9A-D). Consistently, pharmacological inhibition of TRPM2 by CLT or chelating intracellular Ca^2+^ by treatment with BAPTA-AM impeded cisplatin-induced increase in the level of LC3B II in WT MEFs, indicating an inhibitory effect on autophagy activation (Figure 9E-F). In addition, cisplatin-induced downregulation of phosphorylation levels of AKT and p70S6K was also evidently augmented after treatment with CLT or BAPTA-AM, suggesting that TRPM2 activity or, more specifically, TRPM2-mediated Ca^2+^ influx regulates the AKT-mTOR signaling pathway (Figure 9G). Ca^2+^ homeostasis is critical for the activity of mitochondrial respiratory chain, and we therefore investigated whether TRPM2 regulated mitochondrial Ca^2+^. A marked increase in mitochondrial Ca^2+^ level, indicated by an increase in the fluorescence of Rhod-2, was detected in both WT and TRPM2 deficient mRTECs, but there existed no significant difference (Figure 9H-I). Mitophagy is mediated mainly by serine/threonine-protein kinase PINK1 and mitophagy receptor BCL2 and BNIP3 49. The expression levels of both PINK1 and BNIP3 in mRTECs was not affected by TRPM2 silencing (Figure 9J).

### TRPM2 promotes the anti-tumor effect of cisplatin

TRPM2 was shown to facilitate tumor cell proliferation but also contribute to tumor susceptibility to neutrophil cytotoxicity 50. Thus, we further explored whether blocking or enhancing TRPM2 activity influenced the anti-tumor effect of cisplatin. Two known TRPM2 inhibitors, 2-APB and CLT, were tested in human colon cancer cell HCT116, colorectal adenocarcinoma cell SW480 and renal cancer cell ACHN. Treatment with 2-APB Figure S4A-C) and CLT (Figure S4D-F) markedly attenuated cisplatin-induced cytotoxicity in these tumor cell lines, examined by the CCK-8 assays, Conversely, incubation with ADPR, a TRPM2 activator, dramatically increased tumor cell death following treatment with cisplatin (Figure S4G-I). Such ADPR-induced reduction in cell viability was however not observed in mRTECs.

## Discussion and Conclusion

Cisplatin is among the most common drugs causing AKI. To date, there is still unmet demand for effective preventive and therapeutic strategies for cisplatin-induced AKI. In this study, we provide the first line of evidence to suggest that TRPM2 deficiency in mice increases the susceptibility to cisplatin-induced nephrotoxicity through impairing activation of autophagy and disturbing mitochondrial homeostasis.

TRPM2 has been linked to a diversity of oxidative stress-related disorders (16. Acetaminophen overdosing can induce oxidative stress and TRPM2 activation, leading to Ca^2+^ overload and thereby causing hepatocellular death and liver toxicity 22. In ischemic AKI, TRPM2 has been shown to interact with and promote the activation of RAC1, leading to increased NADPH activity and oxidative stress to cause damage to kidneys 30. The present study shows that cisplatin-induced mitochondrial ROS production and oxidative damage in kidneys were exacerbated in TRPM2-deficient mice, suggesting a context-dependent role of TRPM2 in kidneys. Notably, we observed substantial localization of TRPM2 in the cytoplasm of renal tubular cells, particularly in mitochondria, as shown in TRPM2-transfected HEK-293 cells 51. Mitochondria expression of TRPM2 has also been reported in hippocampal neurons 52 and SH-SY5Y neuroblastoma cells 53, but, to date, the exact role of TRPM2 in this intracellular organelle is not fully defined. Considering that only a small part of TRPM2 was presented in mitochondria and its expression was not altered by treatment with cisplatin, it remains possible that TRPM2 regulates cisplatin-induced kidney injury via modulating various organelles, as well as acting as an ion channel on cell surface.

Mitochondria, while representing the major source of intracellular ROS, are the intracellular organelles that are highly vulnerable to damage by ROS. Due to a lack of protective histones, mtDNA is also more susceptible to cisplatin-induced damage than nuclear DNA 54. Therefore, mitochondrial dysfunction is likely responsible for the deterioration of kidney damage in *Trpm2^-/-^* mice. In support of this notion, we showed that cisplatin-induced mitochondrial fragmentation, ROS production, oxidative damage and release of Cyt-c in kidneys, all of which were aggravated by TRPM2 deficiency, were largely prevented after treatment with the mtROS scavenger Mito-TEMPO. Previous studies examining gastric cancer cells and leukemia cells showed that TRPM2 depletion led to mitochondrial dysfunction through impeding mitochondrial protein expression and mitochondrial Ca^2+^ uptake 28, 29. Here, while we found that TRPM2 was present in mitochondria, there were no evident abnormalities in mitochondrial structure and mtDNA content in *Trpm2^-/-^* kidneys and no substantial differences in ROS production and MMP between untreated WT and TRPM2 deficient mRTECs or MEFs. Cisplatin-induced mitochondrial Ca^2+^ uptake appeared to be unaffected by silencing TRPM2. These discrepancies may be explained by the different subcellular distribution and multiple functions of TRPM2 in various tissues or cells, as well as the different ways of silencing TRPM2, as several splice variants with distinct functions have been identified 55. Thus, we speculate that TRPM2 may participate in certain mitochondria quality control mechanism instead of directly influencing mitochondrial function.

It is known that the initiation of caspase-dependent apoptosis in response to cisplatin is preceded by the increase in LC3 II and autophagosome formation during AKI 56. Inhibiting autophagy with CQ aggravates renal tubular cell apoptosis induced by cisplatin 15, while promoting autophagy with metformin has an opposite effect 57. In the present study, cisplatin-induced accumulation of LC3 II and an increase in the number of autolysosomes were observed both *in vitro* and *in vivo*, confirming that autophagy was activated by cisplatin. However, these alterations were markedly attenuated in *Trpm2^-/-^* mice, suggesting a disturbance of autophagy. Furthermore, administration of rapamycin not only restored the autophagy process but also abolished mitochondrial dysfunction, cell apoptosis and kidney injury, implying a critical role of TRPM2-mediated autophagy in defending against cisplatin-induced nephrotoxicity. In gastric cancer cells, TRPM2 depletion impaired the expression of BNIP3, a key mitophagy receptor 29. However, in mRTECs, TRPM2 silencing resulted in no significant decrease in the expression of BNIP3 or PINK1, implying that TRPM2 does not regulate mitophagy directly by these two mechanisms.

Our study revealed that TRPM2 was strongly associated with cisplatin-induced autophagy, though GSEA analysis on autophagy pathway displayed no significant difference between WT and *Trpm2^-/-^* mice Figure S5A). Intriguingly, several lines of evidence suggest a positive role of TRPM2 in ROS-induced autophagy under various conditions, such as nano-ZnO-treated human perivascular cells (58 and epidermal growth factor-treated human corneal epithelial cells 59. There is evidence that TRPM2 activation can also inhibit H_2_O_2_-triggered autophagy through a Ca^2+^-dependent feedback mechanism 43. We found that TRPM2 silencing increased the LC3 II level in response to exposure to H_2_O_2_ in a concentration-dependent manner Figure S5B). Therefore, further investigation is required to better understand the mechanisms underlying the regulation by TRPM2 of different stress-induced autophagy. In this study, we showed that the AKT-mTOR signaling pathway was activated *in vitro* and *in vivo* as a result of TRPM2 silencing. Pharmacological inhibition of AKT or mTOR led to substantial elevation of the LC3 II level and formation of autophagosomes and autolysosomes, supporting that TRPM2 regulates autophagy via modulating the AKT-mTOR pathway. mTOR acts as a typical negative regulator of autophagy via phosphorylating autophagy-initiating kinase ULK1 at Ser 757 and thereby preventing its activation (60. Intensive studies have shown that targeting mTOR effectively attenuated cisplatin-induced kidney injury through improving autophagy 13, 15, 45. AKT could indirectly activate mTOR through inhibition of TSC2, a suppressor of mTOR 61. It has been reported that AKT-mediated inhibition of autophagy could aggravate cisplatin-induced nephrotoxicity 45 and ototoxicity 62.

Our recent work 63 shows that TRPM2 deficiency attenuates cerebral ischemia-reperfusion injury through enhancing autophagy via promoting the activity of AMPK, an upstream suppressor of mTOR. Here, we observed a marked increase in the phosphorylation level of AMPK after treatment with cisplatin, which however remained similar between *Trpm2^-/-^* and WT MEFs Figure S5C), implying that AMPK was not involved in the inhibition of cisplatin-induced autophagy due to TRPM2 deficiency. TRPM2 activation by oxidative stress can phosphorylate beclin1 at Ser295 through a Ca^2+^-dependent mechanism, leading to the inhibition of autophagy (43. In contrast, the phosphorylation level of beclin1 was augmented in cisplatin-treated *Trpm2^-/-^* MEFs Figure S5D). The conflicting findings can be in part explained by a direct interaction of AKT with beclin1 that promotes beclin1 phosphorylation (64.

Numerous studies have shown that intracellular Ca^2+^ homeostasis influences a variety of process, including autophagy. Serval ion channels have the ability to modulate autophagy by controlling intracellular ion concentrations. For example, TRPV2 and TRPV4 can promote autophagy through generating Ca^2+^ signals that block the AKT activity 46, 65. In this study, we sought to elucidate the relationship of TRPM2-mediated Ca^2+^ signal and autophagy activation. TRPM2 silencing blocked cisplatin-induced Ca^2+^ signal in the cytoplasm but not in the mitochondria. Both blockage of the TRPM2 channel and chelation of intracellular Ca^2+^ prevented the accumulation of LC3B II (Figure 9E-F) and, moreover, significantly attenuated cisplatin-induced downregulation of the AKT-mTOR signaling pathway (Figure 9G). Collectively, these findings indicate that TRPM2 mediates autophagy in a Ca^2+^-AKT-mTOR-dependent mechanism. Whether there is another mediator between Ca^2+^ signal and AKT activity merits further investigation.

It has been reported that cisplatin-induced cell death in human laryngeal squamous cancer cells and brain tumor cells was enhanced by treatment with curcumin 66 or eicosapentaenoic acid 67, respectively, via activating TRPM2. However, cell death in normal kidney (MPK) cell was reduced by treatment with curcumin 66. In this study, the anti-tumor effect of cisplatin was weakened by inhibiting TRPM2 with 2-APB and CLT and, conversely, enhanced by activating TRPM2 with ADPR, suggesting that TRPM2 is a potential target for cisplatin-based chemotherapy. It is generally accepted that ADPR is generated by the consecutive actions of poly (ADPR) polymerase and poly (ADPR) glycohydrolase, two main enzymes critically engaged in DNA repair after nuclear DNA being attacked by ROS or DNA-damaging agents, such as cisplatin 16. It is likely that TRPM2 activation augments oxidative stress that sensitizes cancer cell to cisplatin-based chemotherapies. Nonetheless, the clinical translation of TRPM2 agonists is still limited due to several reasons. Firstly, TRPM2 activation has been involved in a variety of diseases, such as ischemia brain injury 20 and liver damage 22, and approaches to avoid these potential side effects are still lacking. Secondly, TRPM2 is critical for proliferation of tumor cells, and TRPM2 activation increases the risks of tumor progression 68. Thirdly, ADPR is implicated in ADP-ribosylation, a post-translational modification that plays a pivotal role in a wide variety of cellular biological events 69. How to overcome these disadvantages of TRPM2 agonists merits further investigations. Here, we also found that addition of ADPR failed to augment the level of autophagy and protect mRTECs against cisplatin-induced injury (Figure S6A-C). We surmised that cisplatin-induced generation of ADPR was sufficient to provoke autophagy through activating TRPM2 channel, or perhaps that the protective effect of extra ADPR was offset by a rise in oxidation caused by itself. Similarly, overexpression of TRPM2 could neither facilitate the induction of autophagy nor boost the formation of autophagosomes and autolysosomes induced by cisplatin (Figure S6D-F). It is possible that the basal level of TRPM2 activity upon cisplatin treatment was sufficient to initiate the downstream autophagic response for the protection of tubular cells. In the future, it is important to gain more insights into the mechanisms by which TRPM2 determines cell fates in different genetic background and such information is essential for developing targeted and individualized treatments.

In summary, our study is the first to show that TRPM2 is important in protecting cisplatin-induced AKI via the AKT-mTOR signaling pathway and autophagy. Our findings provide novel insights into the mechanism of autophagy regulation and highlight the multifaceted roles of TRPM2 under various pathogenic conditions.

## Supplementary Material

Supplementary figures.

## Abbreviations

ADPR: adenosine diphosphate-ribose
AKI: Acute kidney injury
AKT: protein kinase B
BNIP3: adenovirus E1B 19-kDa-interacting protein 3
CLT: Clotrimazole
CQ: chloroquine
Cyt: cytochrome
DHE: Dihydroethidium
HBSS: Hank's Balanced Salt Solution
Kim-1: kidney injury marker-1
LAMP: lysosomal associated membrane protein
LTL: Lotus tetragonolobus lectin
MEFs: mouse embryonic fibroblasts
MMP: mitochondrial membrane potential
mRTECs: mouse renal tubular epithelial cells
mtDNA: mitochondrial DNA
mTOR: mammalian target of rapamycin
mtROS: mitochondrial ROS
OCT: optimal cutting temperature
PAF: paraformaldehyde
PAS: Periodic Acid-Schiff
ROS: reactive oxygen species
TEM: Transmission electron microscopy
TRPM2: Transient receptor potential melastatin 2
3-MA: 3-methyladenine
