# Supplementary material for: TRPM2 protects against cisplatin-induced acute kidney injury and mitochondrial dysfunction via modulating autophagy
Source: Theranostics. 2023 Jul 31;13(13):4356–75. doi: 10.7150/thno.84655 (PMC10465213; doi:10.7150/thno.84655)
Supplement: Supplementary file 1 — Supplementary figures. [file thnov13p4356s1.pdf]

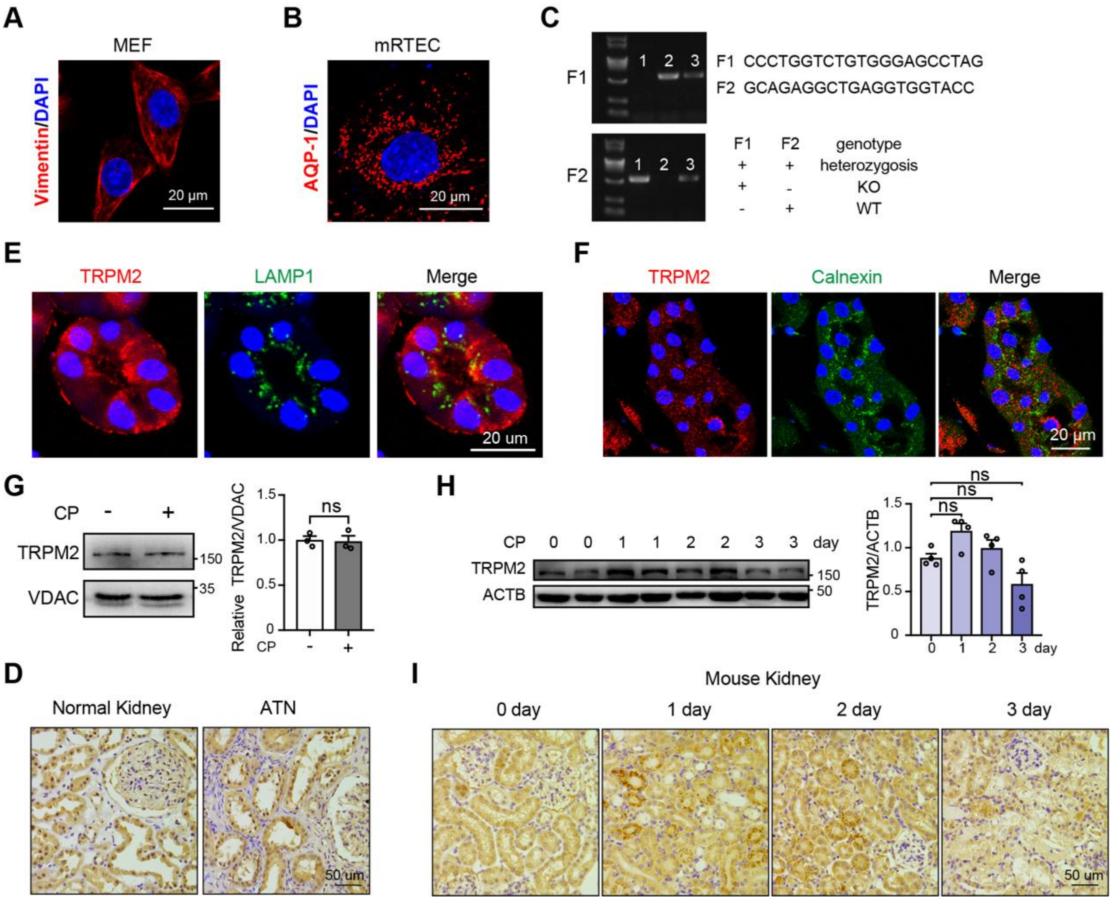

Figure S1. Validation and distribution of TRPM2 in tubular epithelial cells and in kidneys. (A) Representative confocal images of primary MEFs stained with fibroblast marker Vimentin. Scale bars, 20 µm. **(B)** Representative confocal images of primary mRTECs stained with tubular epithelial marker AQP-1. Scale bars, 20 µm. **(C)** Gene validation of TRPM2 knockout. **(D)** Immunohistochemistry of TRPM2 in normal kidney tissues adjacent to tumor and in injured kidneys pathologically characterized by acute tubular necrosis (ATN). Scale bars, 50 µm. **(E)** Representative confocal images of renal tubular cells double-labeled TRPM2 and lysosomal marker LAMP1 in normal kidneys. Scale bars, 20 µm. **(F)** Representative confocal images of renal tubular cells double-labeled TRPM2 and endoplasmic reticulum marker Calnexin in normal kidneys. Scale bars, 20 µm. **(G)** Immunoblotting analysis and quantification of TRPM2 in mitochondrial extracts of mRTECs with or without 5 µM cisplatin (CP) intervention for 24 h. **(H, I)** Immunoblotting analysis and immunohistochemistry of TRPM2 in kidneys following treatment with 18 mg/kg CP for 0, 1, 2 and 3 days. Data are presented as mean ± SEM. Statistical analysis was performed using one-way ANOVA with Tukey post-hoc test or two-tailed unpaired Student's t-test. ns, not significant; *$p < 0.05$, **$p < 0.01$, ***$p < 0.001$.

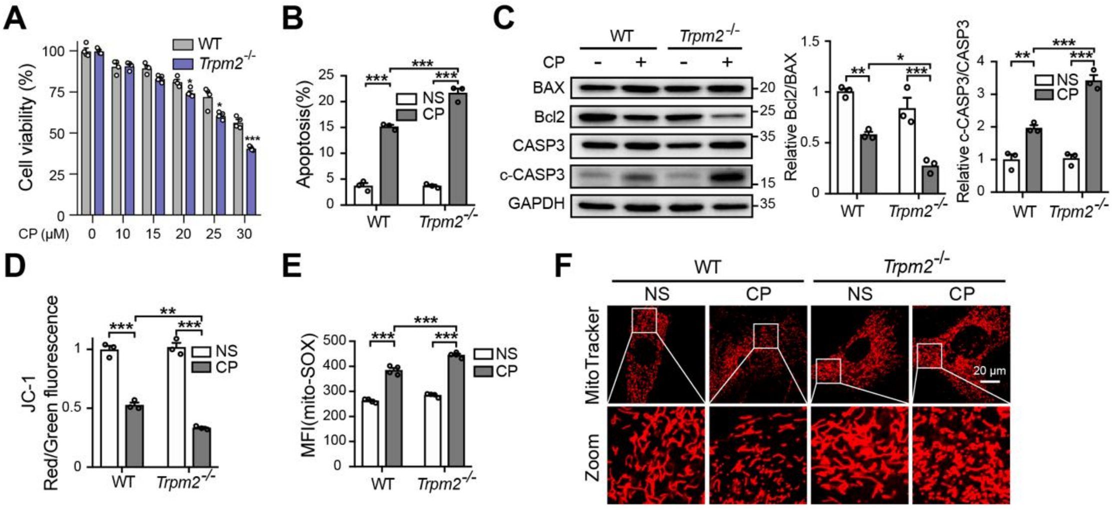

Figure S2. TRPM2 knockout exacerbates apoptosis in MEFs subjected to cisplatin. (A) Cell viability of WT and *Trpm2*$^{-/-}$ MEFs incubated with cisplatin (CP) at indicated concentrations for 24 h (n = 3). Then, WT and *Trpm2*$^{-/-}$ MEFs were treated with 20 μM cisplatin for 24 h. **(B)** Cell apoptosis determined by flow cytometry (n = 3). **(C)** Immunoblotting analysis and quantification of BAX, Bcl2, cleaved and total caspase-3 (CASP3). **(D)** Mitochondrial membrane potential indicated by the ratio of red to green fluorescence intensity of JC-1 (n = 3). **(E)** Level of mitochondrial ROS determined by the mean fluorescence intensity (MFI) of Mito-SOX (n = 4). **(F)** Mitochondrial morphology detected by the fluorescence of MitoTracker Red. Scale bars, 20 μm. Data are presented as mean ± SEM. Statistical analysis was performed using one-way ANOVA with Tukey post-hoc test. *$p < 0.05$, **$p < 0.01$, ***$p < 0.001$.

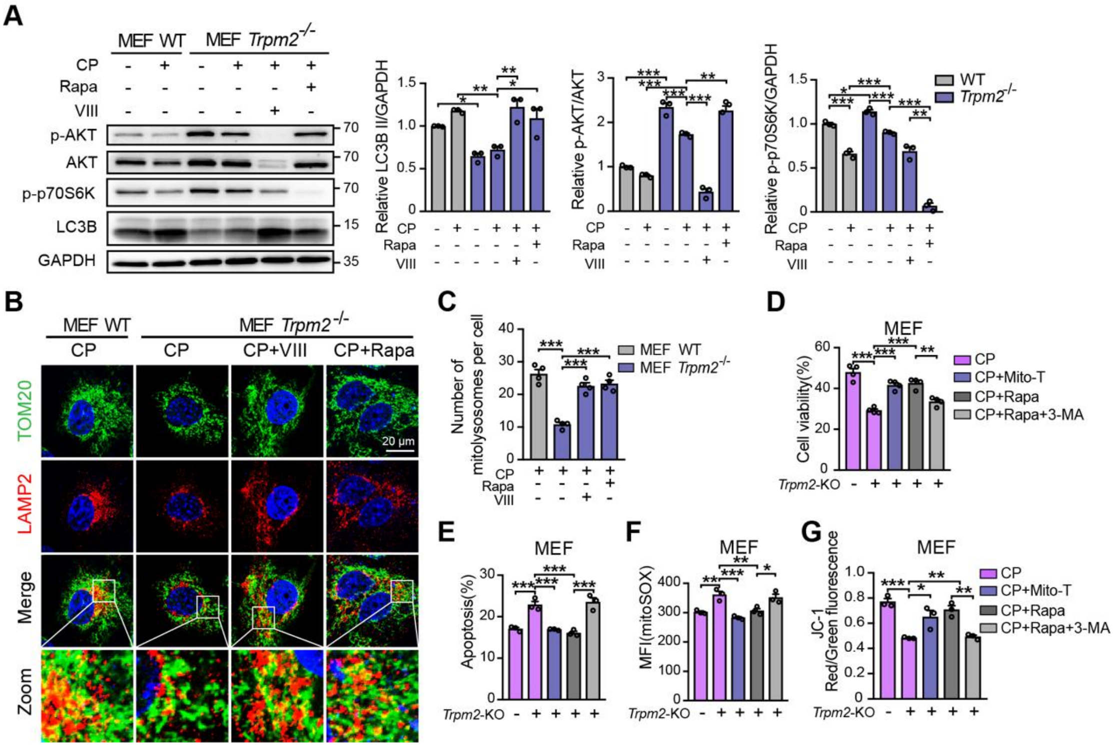

Figure S3. TRPM2 regulates cisplatin-triggered autophagy via suppressing AKT-mTOR signaling in MEFs. *Trpm2-/-* MEFs were pre-incubated with AKT inhibitor VIII (5 μM) or mTOR inhibitor rapamycin (Rapa, 50 nM) for 1 h followed by 20 μM cisplatin (CP) intervention for 24 h. **(A)** Immunoblotting analysis and quantification of LC3B II, p-AKT and p-p70S6K in MEFs following treatment with 20 μM CP for 24 h. **(B, C)** Representative confocal images and quantification of mitolysosomes evaluated by immunofluorescence double-labeled TOM20 and LAMP2 in MEFs (n = 4). Scale bars, 20 μm. Then, *Trpm2-/-* MEFs were pre-incubated with rapamycin (50 nM), Mito-TEMPO (Mito-T, 200 nM) or Rapamycin (50 nM) plus 3-MA (5 mM) for 1 h followed by treatment with 20 μM CP for 24 h. **(D)** Cell viability of MEFs determined by CCK-8 assay (n = 4). **(E)** CP-induced apoptosis in MEFs determined by flow cytometry (n = 3). **(F)** Level of mitochondrial ROS in MEFs determined by the mean fluorescent intensity (MFI) of Mito-SOX (n = 3). **(G)** Mitochondrial membrane potential of MEFs indicated by the ratio of red to green fluorescence intensity of JC-1 (n = 3). Data are presented as mean ± SEM. Statistical analysis was performed using one-way ANOVA with Tukey post-hoc test. *$p < 0.05$, **$p < 0.01$, ***$p < 0.001$.

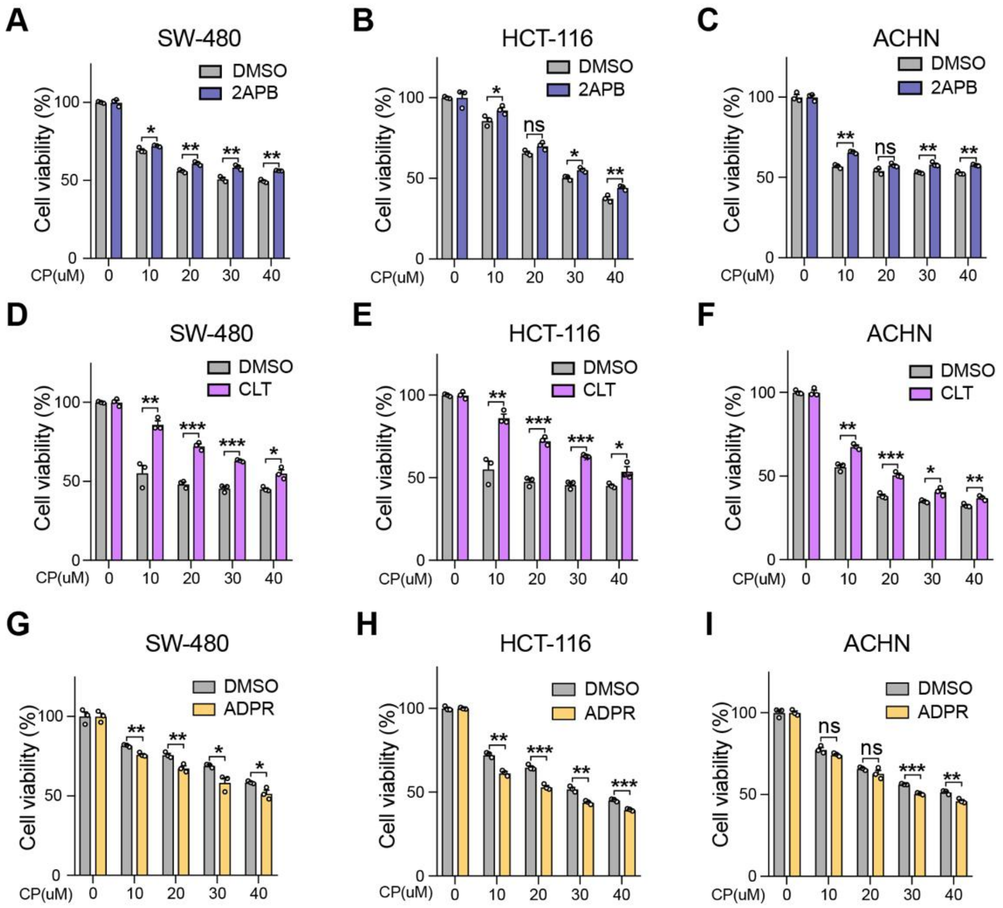

Figure S4. TRPM2 contributes to cisplatin-induced cytotoxicity in several cancer cell lines. Cell viability of SW-480 **(A)**, HCT-116 **(B)** and ACHN **(C)** incubated with TRPM2 inhibitor 2-APB (20 μM) or DMSO for 1 h followed by the treatment with cisplatin (CP) at the indicated concentrations for 24 h (n = 3). Cell viability of SW-480 **(D)**, HCT-116 **(E)** and ACHN **(F)** incubated with TRPM2 inhibitor clotrimazole (CLT, 20 μM) or DMSO for 1 h followed by the treatment with CP at the indicated concentrations for 24 h (n = 3). Cell viability of SW-480 **(G)**, HCT-116 **(H)** and ACHN **(I)** incubated with TRPM2 activator ADPR (100 μM) or DMSO for 1 h followed by treatment with CP at indicated concentrations for 24 h (n = 3). Data are presented as mean ± SEM. Statistical analysis was performed using two-tailed unpaired Student's t-test. ns, not significant; *$p < 0.05$, **$p < 0.01$, ***$p < 0.001$ versus DMSO group.

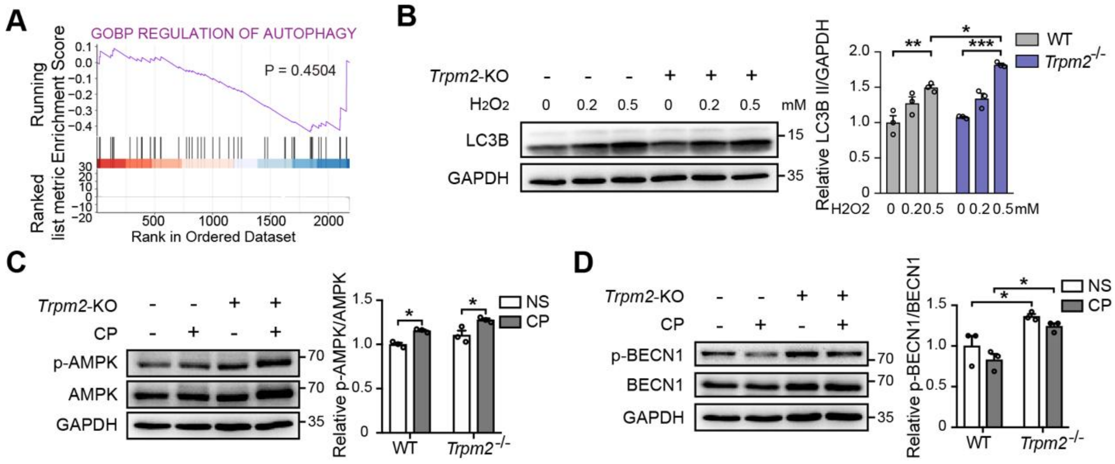

Figure S5. TRPM2 plays context-sensitive roles in the regulation of autophagy. (A) Gene Set Enrichment Analysis of autophagy pathway generated from *Trpm2*$^{-/-}$ and WT mice subjected to cisplatin. **(B)** Immunoblotting analysis and quantification of LC3B II in *Trpm2*$^{-/-}$ and WT MEFs following treatment with H_2O_2 at the indicted concentrations for 3 h. Immunoblotting analysis and quantification of phosphorylated and total **(C)** AMPK and **(D)** beclin1 (BECN1) in MEFs treated with 20 μM cisplatin (CP) or normal saline (NS) for 24 h. Data are presented as mean ± SEM. Statistical analysis was performed using one-way ANOVA with Tukey post-hoc test. *p < 0.05, **p < 0.01, ***p < 0.001.

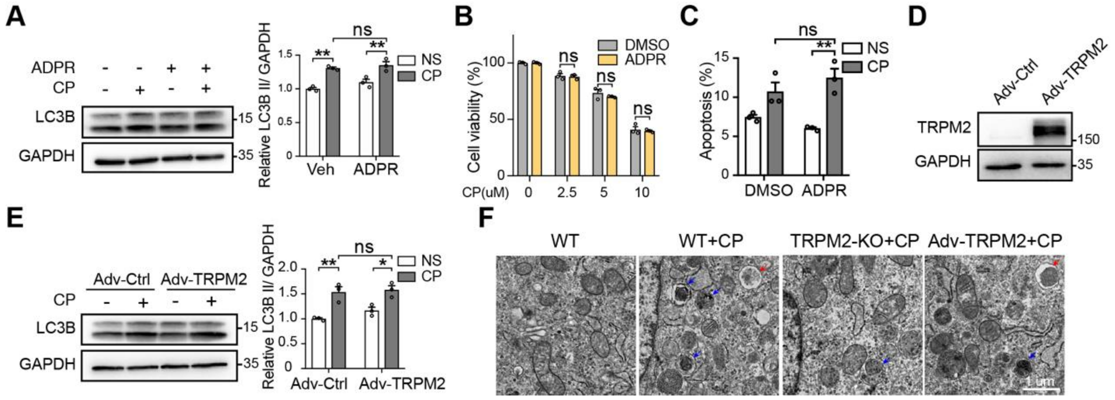

Figure S6. Activation and overexpression of TRPM2 play no protective roles in the cytotoxicity of cisplatin. (A) Immunoblotting analysis and quantification of LC3B II in mRTECs incubated with TRPM2 agonist ADPR (100 μM) or DMSO for 1 h followed by the treatment with 5 μM cisplatin (CP) for 24 h. **(B)** Cell viability of mRTECs incubated with TRPM2 agonist ADPR (100 μM) or DMSO for 1 h followed by the treatment with CP at the indicated concentrations for 24 h (n = 3). **(C)** Cell apoptosis determined by flow cytometry (n = 3). **(D)** Immunoblotting images showing overexpression of TRPM2 using adenovirus (Adv) in mRTECs. **(E)** Immunoblotting analysis and quantification of LC3B II in mRTECs infected with control or TRPM2-overexpressing adenovirus before incubation with 5 μM CP for 24 h. **(F)** Representative TEM images showing the formation of autophagosomes (red arrow) and autolysosomes (blue arrow) in mRTECs treated with 5 μM CP for 24 h. Data are presented as mean ± SEM. Statistical analysis was performed using one-way ANOVA with Tukey post-hoc test. ns, not significant; *p < 0.05, **p < 0.01, ***p < 0.001.
